# Loss of UCP1 function augments recruitment of futile lipid cycling for thermogenesis in murine brown fat

**DOI:** 10.1016/j.molmet.2022.101499

**Published:** 2022-04-22

**Authors:** Josef Oeckl, Petra Janovska, Katerina Adamcova, Kristina Bardova, Sarah Brunner, Sebastian Dieckmann, Josef Ecker, Tobias Fromme, Jiri Funda, Thomas Gantert, Piero Giansanti, Maria Soledad Hidrobo, Ondrej Kuda, Bernhard Kuster, Yongguo Li, Radek Pohl, Sabine Schmitt, Sabine Schweizer, Hans Zischka, Petr Zouhar, Jan Kopecky, Martin Klingenspor

**Affiliations:** 1 Chair for Molecular Nutritional Medicine, TUM School of Life Sciences, Technical University of Munich, Freising, Germany; 2 EKFZ - Else Kröner Fresenius Center for Nutritional Medicine, Technical University of Munich, Freising, Germany; 3 ZIEL Institute for Food & Health, Technical University of Munich, Freising, Germany; 4 Chair of Proteomics and Bioanalytics, TUM School of Life Sciences, Technical University of Munich, Freising, Germany; 5 Bavarian Center for Biomolecular Mass Spectrometry, Technical University of Munich, Freising, Germany; 6 Institute of Toxicology and Environmental Hygiene, School of Medicine, Technical University of Munich, Munich, Germany; 7 Institute of Molecular Toxicology and Pharmacology, Helmholtz Center Munich, Munich, Germany; 8 Laboratory of Adipose Tissue Biology, Institute of Physiology of the Czech Academy of Sciences, Czech Republic; 9 NMR spectroscopy, Institute of Organic Chemistry and Biochemistry of the Czech Academy of Sciences, Czech Republic; 10 Laboratory of Metabolism of Bioactive Lipids, Institute of Physiology of the Czech Academy of Sciences, Czech Republic

**Keywords:** Brown adipose tissue, UCP1-independent thermogenesis, Futile substrate cycle, Lipolysis, Re-esterification, Fatty acids

## Abstract

**Objective:**

Classical ATP-independent non-shivering thermogenesis enabled by uncoupling protein 1 (UCP1) in brown adipose tissue (BAT) is activated, but not essential for survival, in the cold. It has long been suspected that futile ATP-consuming substrate cycles also contribute to thermogenesis and can partially compensate for the genetic ablation of UCP1 in mouse models. Futile ATP-dependent thermogenesis could thereby enable survival in the cold even when brown fat is less abundant or missing.

**Methods:**

In this study, we explore different potential sources of UCP1-independent thermogenesis and identify a futile ATP-consuming triglyceride/fatty acid cycle as the main contributor to cellular heat production in brown adipocytes lacking UCP1. We uncover the mechanism on a molecular level and pinpoint the key enzymes involved using pharmacological and genetic interference.

**Results:**

ATGL is the most important lipase in terms of releasing fatty acids from lipid droplets, while DGAT1 accounts for the majority of fatty acid re-esterification in UCP1-ablated brown adipocytes. Furthermore, we demonstrate that chronic cold exposure causes a pronounced remodeling of adipose tissues and leads to the recruitment of lipid cycling capacity specifically in BAT of UCP1-knockout mice, possibly fueled by fatty acids from white fat. Quantification of triglyceride/fatty acid cycling clearly shows that UCP1-ablated animals significantly increase turnover rates at room temperature and below.

**Conclusion:**

Our results suggest an important role for futile lipid cycling in adaptive thermogenesis and total energy expenditure.

## Highlights

• A futile ATP-consuming triglyceride/fatty acid cycle is main contributor to heat production in brown adipocytes lacking UCP1.
• During active lipolysis, ATGL releases fatty acids from lipid droplets and DGAT1 mediates fatty acid re-esterification.
• UCP1-ablated animals significantly increase triglyceride/fatty acid cycling rates at room temperature and below.

## 1 Introduction

A futile cycle is defined as at least two opposing metabolic pathways being active at the same time, where the forward reaction is competing with an enzyme or a set of enzymes catalyzing the reverse reaction. Since the involved reactions are not operating at a near-equilibrium, substrate flux through a futile cycle will always require the dissipation (informal: “loss”) of chemical energy as heat. The term “futile cycle” was coined, because one means of converting chemical energy into heat is the hydrolysis of ATP, which in the first place does not seem to be a very favorable concept [1]. However, the utility of substrate cycles, instead of their futility, was immediately foregrounded, as soon as the first beneficial aspects were discovered. Futile cycles can contribute to non-shivering thermogenesis (NST), confer precise metabolic control, and expand metabolic flexibility in an energetically challenging setting [[2], [3], [4], [5]].

Futile substrate cycles are a long-standing concept and various examples have been showcased in an array of different invertebrate and vertebrate species. Several glycolytic substrate cycles in insects [[6], [7], [8]], a futile calcium (Ca^2+^) cycle in the heater organ of a large pelagic predatory fish, the blue marlin [9], an extracellular inter-organ lipid cycle between liver and adipose tissue as well as an intracellular adipocyte-specific triglyceride/fatty acid (TG/FA) cycle in rodents and humans [[10], [11], [12], [13], [14], [15], [16], [17], [18], [19], [20]], futile substrate cycling between *de novo* FA synthesis and FA oxidation in skeletal muscle of mice [21], and futile Ca^2+^ cycling mediated by sarcoplasmic reticulum calcium ATPase (SERCA) pump activity in skeletal muscle of mammals, reviewed in [22]. For thermogenic adipocytes, there has been a recent revival of interest in futile substrate cycles [23], since ATP driven cyclic Ca^2+^ pumping [24] and cyclic interconversion of creatine/creatine-phosphate [25] were discovered as contributors to NST in murine adipose tissues and the control of whole-body energy homeostasis.

Mice and other small mammals heavily rely on UCP1-mediated NST in the cold [26], and thus the unexpected cold resistance of UCP1-knockout (UCP1KO) mice has always been and still is an unresolved mystery inextricably intertwined with alternative means of thermogenesis and futile substrate cycles [[27], [28], [29], [30], [31]]. Two recent studies have very convincingly shown that UCP1KO mice increase whole body thermogenesis and specifically interscapular BAT (iBAT) temperature following adrenergic stimulation [32,33], yet the futile reactions mediating thermogenesis in the absence of UCP1 remain unknown. In the light of the limited capacity of human brown adipose tissue for UCP1-dependent thermogenesis, resolving the nature of these futile cycles is of primary interest, as they may play a more important role for human energy balance [16,34] than mostly assumed so far. In this study, we explored different UCP1-independent thermogenic pathways in brown adipocytes of UCP1KO mice and their contribution to cellular heat production. We analyzed changes in the proteome of wild type (WT) and UCP1KO adipocytes during acute adrenergic stimulation to detect characteristic molecular signatures, which allowed us to narrow down our search to potential candidates related to Ca^2+^ and lipid metabolism. In the next step, we specifically targeted these putative futile cycles by means of genetic and pharmacological perturbations to pinpoint the involved enzymes, and corresponding pathways. A futile cycle of lipolysis and re-esterification of FAs emerged as the predominant source of UCP1-independent thermogenesis in brown UCP1KO adipocytes, and therefore we hypothesized that UCP1KO mice would primarily recruit lipid cycling capacity to aid in maintaining normothermia. Finally, we attempted to quantify futile lipid cycling in the adipose tissue of WT and UCP1KO mice at different temperatures, and indeed observed strong genotype-dependent differences with higher cycling rates in UCP1KO mice. These results demonstrate that a futile ATP-consuming cycle of TG lipolysis and FA re-esterification contributes to NST in brown adipocytes of UCP1KO mice and facilitates their survival in the cold.

## 2 Material and methods

### 2.1 Animals

#### 2.1.1 Animals for cell culture

129Sv/S1 UCP1KO^+/-^ mice were bred at the animal facility of the Technical University of Munich as approved by the Government of Upper Bavaria and in accordance with the German Animal Welfare Act. All mice were kept under controlled housing conditions (55% relative humidity, 23 °C ambient temperature, 12 h/12 h light dark cycle) and had *ad libitum* access to food and water. Homozygous UCP1KO and WT mice were used for the isolation of primary pre-adipocytes (see 2.4). This animal model was developed by Dr. Leslie P. Kozak [26].

#### 2.1.2 Animals for analysis of brown adipose tissue proteome

WT and UCP1KO mice on a C57BL/6N background were generated as described previously [35]. All animals were bred in a specific pathogen-free facility under controlled housing conditions (55% relative humidity, 23 °C ambient temperature, 12 h/12 h light dark cycle) and had *ad libitum* access to food and water. The experiments were performed according to the German Animal Welfare Act with permission from the Government of Upper Bavaria (Regierung von Oberbayern, reference number ROB-55.2-2532.Vet_02-16-166). At the age of 8-weeks, male homozygous UCP1KO and WT mice were single caged, transferred to climate cabinets set to 23 °C and 55% relative humidity. Simultaneously, mice were switched to a control diet (Snifff Cat. No S5745-E702) and divided into two groups based on body weight. Animals were acclimated to cold as followed. After 1 week at 23 °C, an experimental group (5 °C) was gradually acclimatized to cold by decreasing the temperature to 20 °C, 15 °C, 10 °C, and finally 5 °C for 1 week, whereas a control group was kept at 23 °C (23 °C) for 4 weeks. All mice were killed by CO_2_ asphyxiation and iBAT was dissected, weighed, and immediately snap frozen in liquid nitrogen. Tissues were stored at −80 °C until further processing.

#### 2.1.3 Animals for *in vivo* evaluation of TG synthesis and DNL

Homozygous UCP1KO mice and their WT littermates with C57BL/6J genetic background were used. These mice were derived from heterozygous breeding pairs at the animal facility of the Institute of Physiology of the Czech Academy of Sciences, Prague, Czech Republic, where the animal experiments were conducted. This animal model was developed by Dr. Leslie P. Kozak [26] and imported to Prague through the Technische Universität München, Freising, Germany.

Mice were born and maintained at either 30 °C or 20 °C, 50% humidity, 12 h/12 h light/dark cycle (light from 6 a.m.), with drinking water and standard chow diet (extruded ssniff R/M−H from Ssniff Spezialdiaten GmbH, Soest, Germany; metabolizable energy 13 MJ/kg) *ad libitum*. They were weaned at 4 weeks of age. At 9 weeks of age, male mice were single caged and randomly assigned to a 6-week-acclimation to (i) warm (WA, 30 °C); (ii) moderate cold (MCA, 20 °C); and (iii) cold (CA, 6 °C). CA mice were kept first 3 weeks at 20 °C, then another 3 weeks at 6 °C. Animals were killed in a non-fasted state by cervical dislocation under diethyl ether anaesthesia (between 8 and 10 a.m.). Liver, heart, epididymal WAT (eWAT), inguinal (iWAT), iBAT and quadriceps muscle were dissected and tissue samples were snap frozen in liquid nitrogen, EDTA-plasma was collected, and all the samples were stored at −80 °C. The experiments followed the guidelines for the use and care of laboratory animals of the Institute of Physiology of the Czech Academy of Sciences and were approved under protocol no. 48/2019.

### 2.2 Biochemical analysis of plasma and tissue samples

Plasma levels of TGs and total cholesterol were determined using the colorimetric enzymatic assays from Erba Lachema (Brno, Czech Republic), and non-esterified fatty acids were assessed with a NEFA-HR(2) kit from Wako Chemicals GmbH (Neuss, Germany). Blood glucose levels were measured by OneTouch Ultra glucometers (LifeScan, Milpitas, CA, USA) and plasma insulin levels were determined by the Sensitive Rat Insulin RIA Kit (Merck Millipore, Billerica, MA, USA). Tissue TG content was estimated in ethanolic KOH tissue solubilisates as before [36].

### 2.3 In vivo evaluation of TG synthesis and *de novo* lipogenesis (DNL)-derived FAs in eWAT and iBAT

TG synthesis and DNL were characterized using *in vivo* ^2^H enrichment of TGs similarly as before [17]. Two days prior to dissection, mice were injected intraperitoneally with a bolus of ^2^H_2_O in saline (3.5 ml per 100 g body weight) and 5% of their drinking water was replaced by ^2^H_2_O for the rest of the experiment in order to obtain strable 5% ^2^H2O content in body water. After dissection, lipids from eWAT and iBAT were extracted as in FAHFA in [37], except that the samples were homogenized in a mixture of citric acid and ethylacetate. Dried organic phase was resuspended in hexane and applied on Discovery DSC-Si SPE tubes (52 μm, 72 Å; Merck, Darmstadt, Germany). TG fraction was eluted from SPE tubes with a mixture of hexane and MTBE. Samples were analyzed using nuclear magnetic resonance (NMR) spectroscopy.

^1^H and ^2^H NMR spectroscopy was performed as before [17,18] using AVANCE III HD 500 MHz system (Bruker Corporation) equipped with ^19^F lock and a 5-mm CP BBO-^1^H&^19^F–^2^H probe. The spectra were analyzed using MestReNova and spectral deconvolution was used in case of ^2^H for integration of signals. The amount of ^1^H and ^2^H in both glycerol and fatty-acyl moiety of TGs was calculated from the peak area relative to the peak of the pyrazine ^1^H/^2^H standard. Since ^2^H can be incorporated into glycerol moiety of TGs only before esterification of FAs to glycerol, and glycerol formed during lipolysis in WAT is assumed to be released into the circulation and not converted to glycerol-3-phosphate *in situ* [38], TG positional ^2^H enrichment of the glycerol moiety reflects the rate of TG synthesis. Enrichment of newly synthesized glycerol-3-phosphate from ^2^H_2_O is assumed to be stoichiometric for all five positional hydrogens regardless of the relative contributions of glycolysis and glyceroneogenesis [39]. Similarly, ^2^H enrichment of FA methyls in TGs correlates with DNL rate. Measurement of fractional TG/FA cycling draws on previously validated assumptions of glycerol ^2^H-enrichment from body ^2^H-enriched water [39].

### 2.4 Isolation of primary pre-adipocytes and differentiation into adipocytes

Stromal vascular fraction (SVF) was isolated from iBAT of 5–7 week old WT and UCP1KO mice as previously described [40]. Mice were euthanized by CO_2_-asphyxiation and iBAT was dissected. Adipose tissue was mechanically minced followed by enzymatic digestion (collagenase) under constant agitation. Large cell debris and undigested pieces of tissue were removed from the homogenate by filtration (250 μm mesh). SVF was separated from mature adipocytes by centrifugation. Contaminating erythrocytes were lysed with an NH_4_Cl-based buffer. SVF containing pre-adipocytes was pelleted, washed, resuspended in growth medium (DMEM supplemented with 20% FBS, antibiotics, and an antifungal agent), and filtered through a 40 μm cell strainer prior to plating. Growth medium was replaced every other day. When cells reached 80–100% confluency (day 0) growth medium was replaced with induction medium (DMEM supplemented with 10% FBS, antibiotics, 5 μg/ml insulin, 1 nM T3, 125 μM indomethacin, 500 μM IBMX, 1 μM dexamethasone, and 1 μM rosiglitazone). After 48 h (day 2) induction medium was removed and differentiation medium (DMEM supplemented with 10% FBS, antibiotics, 5 μg/ml insulin, 1 nM T3, and 1 μM rosiglitazone) was added. Differentiation medium was replaced every other day for 6 days until cells were considered as fully differentiated adipocytes (day 8). SVF isolated from iBAT and differentiated into mature adipocytes was considered as brown adipocytes.

### 2.5 siRNA-mediated gene silencing

Reverse transfection with DsiRNA or Negative Control DsiRNA (Integrated DNA Technologies) was carried out on day 7 of the differentiation procedure as previously published [40]. 25 μl of transfection mix (OptiMEM supplemented with 30 μl/ml Lipofectamine™ RNAiMAX and 300 nM DsiRNA) per well were added into a Seahorse XF96 cell culture plate and incubated for 20 min at room temperature. Differentiated adipocytes were detached with trypsin and collagenase. Adipocytes were spun down and resuspended in an appropriate volume of differentiation medium (without antibiotics). 125 μl of cell suspension was added to each well of a Seahorse XF96 cell culture plate. After 24 h medium was removed and fresh differentiation medium (containing antibiotics) was added. After another 48 h cells were subjected to oxygen consumption analysis (day 10). RNA was isolated from cells following the respiration assay and knockdown efficiency was determined by qPCR.

### 2.6 Microplate-based respirometry

Cellular oxygen consumption was measured at 37 °C with an XF24 or XF96 Extracellular Flux Analyzer as previously published [40]. Adipocytes were assayed on day 8 (regular culture conditions) or day 10 (DsiRNA-mediated knockdown). On the day of the assay differentiation medium was removed, cells were washed twice with respiration base medium (DMEM base, Sigma–Aldrich D5030, supplemented with 25 mM glucose, 2 mM GlutaMAX™, 31 mM NaCl and 15 mg/l phenol red), and, if not otherwise stated, respiration assay medium (respiration base medium supplemented with 20 mg/ml essentially fatty acid-free BSA) was added to a final volume of 180 μl per well (inhibitors were already added to the assay medium and were present in the medium during the measurement, see 2.8 “Chemicals and inhibitors”). Cells were incubated for 1 h at 37 °C in a laboratory non-CO_2_ incubator prior to the measurement. One measurement cycle consisted of a 4 min “Mix”, no “Wait”, and a 2 min “Measure” period. If not otherwise stated, injections were added in the following order: Isoproterenol (Iso, 100 nM), oligomycin (Oligo, 5 μM), carbonyl cyanide-4-(trifluoromethoxy)phenylhydrazone (FCCP, 7.5 μM), and antimycin A (AA, 5 μM). Concentrations are final concentrations in wells. Since extracellular acidification rate (ECAR) is not a suitable measure of glycolytic flux of adipocytes during active lipolysis [40], we did not include these data. OCR metrics were calculated as follows:

Figure 2A:

$$\text{Oligomycin}-\text{sensitive}\;\text{part}\text{ of Isoproterenol}-\text{stimulated OCR}\;\left(\%\right)=\frac{\left(maximum\;after\;isoproterenol-minimum\;after\;oligomycin\right)}{\left(maximum\;after\;isoproterenol-minimum\;prior\;to\;isoproterenol\right)}*100$$

**Figure 1:**
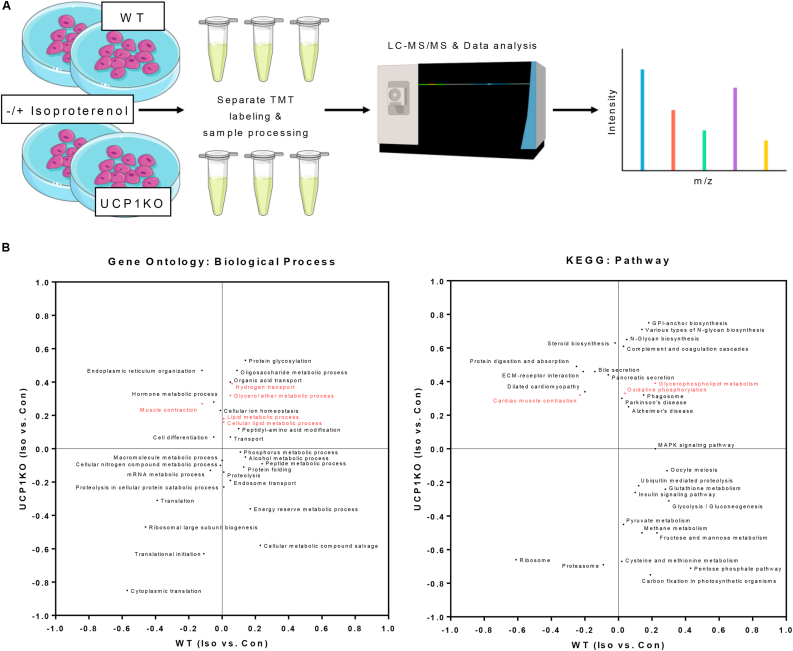
Proteins associated with futile calcium and lipid cycling are acutely upregulated during active lipolysis in brown UCP1-knockout adipocytes. A) Schematic depiction of sample generation, processing, and subsequent proteome analysis. Primary cultures of fully differentiated 129Sv/S1 brown wild type (WT) and UCP1-knockout (UCP1KO) adipocytes were stimulated with 500 nM isoproterenol (Iso) for 30 min. WT and UCP1KO samples were processed and analyzed separately. Pathway enrichment comparing Iso versus no treatment (Con) was performed within each genotype. The complete set of processed mass spectrometry data can be found in Supp. Table 2. B) Two-dimensional annotation enrichment analysis showing biological processes and pathways, which are significantly regulated upon adrenergic stimulation in at least one of the two genotypes. Terms that are preferentially upregulated in stimulated UCP1KO adipocytes and downregulated or not affected in WT cells are located above the x-axis and near or to the left of the y-axis. Values between 0 < x ≤ 1 indicate an upregulation after the addition of isoproterenol, whereas values between −1 ≤ x < 0 indicate a downregulation. Terms related to futile substrate cycles are highlighted in red. Not all terms are displayed due to overlapping points. The complete set of pathways can be found in Supp. Table 3. n = 5 independent biological experiments.

**Figure 2:**
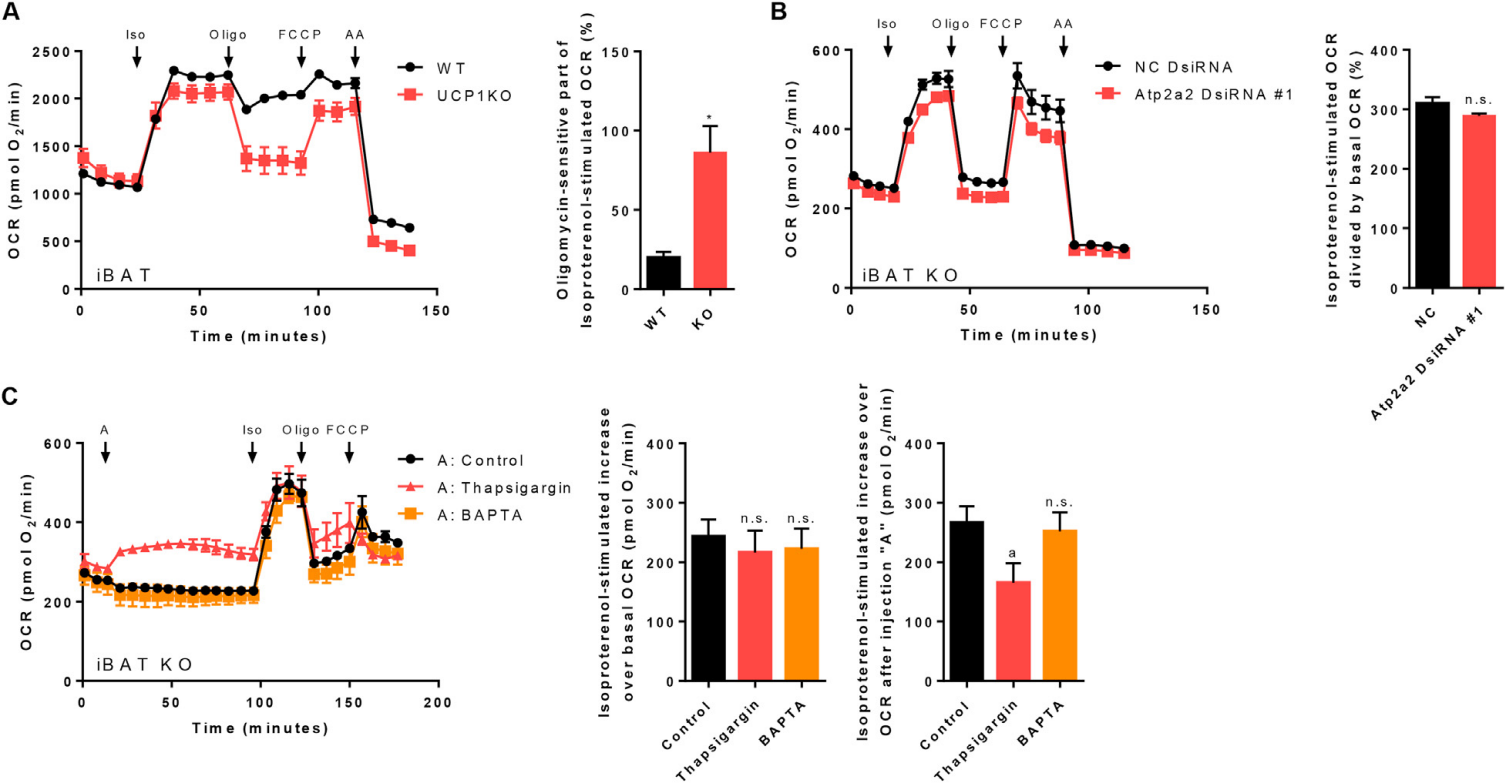
Futile calcium cycling does not contribute to cellular thermogenesis in murine brown UCP1-knockout adipocytes. A) Seahorse XF24 extracellular flux measurement of primary cultures of fully differentiated 129Sv/S1 brown wild type (WT) and UCP1-knockout (UCP1KO) adipocytes and quantification of the oligomycin-sensitive part of stimulated oxygen consumption rate (OCR). n = 10–12 wells from two independent biological experiments. A two-tailed Welch-test was applied. Asterisk (∗) indicates a significant difference between the two groups. Isoproterenol (Iso), oligomycin (Oligo), carbonyl cyanide-4-(trifluoromethoxy)phenylhydrazone (FCCP), antimycin A (AA). B) & C) XF96 extracellular flux measurements of primary cultures of fully differentiated 129Sv/S1 brown UCP1KO adipocytes. B) Atp2a2 was knocked down in brown UCP1KO adipocytes with DsiRNA as described in “Material & Methods” and the isoproterenol-induced increase in OCR was calculated. n = 17–25 wells from three independent biological experiments. A two-tailed t-test was applied. Not statistically significant (n.s.). C) Brown UCP1KO adipocytes were acutely treated with thapsigargin (5 μM final) or BAPTA (20 μM final) for 1 h and the response to isoproterenol was monitored (left). Compounds were added as an injection *via* port “A”. The isoproterenol-induced increase in OCR over basal OCR (middle) and over OCR after the addition of compounds (right) was calculated. n = 18–23 wells from three independent biological experiments. One-way ANOVA followed by Tukey's HSD was applied. “a” indicates a significant difference from the control group. Not statistically significant (n.s.).

**Figure 3:**
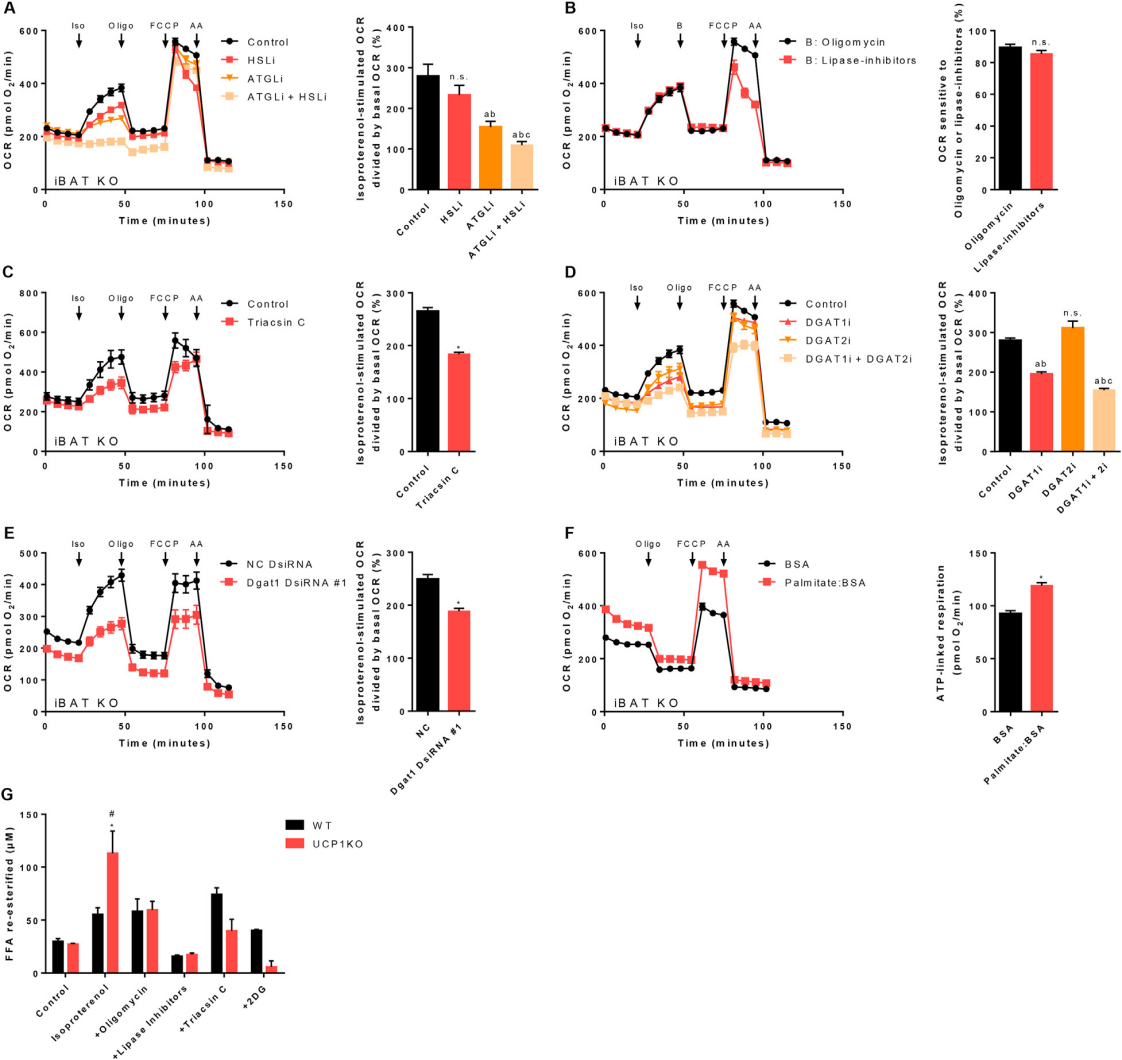
A futile cycle of lipolysis and re-esterification of fatty acids mediates non-shivering thermogenesis in brown UCP1-knockout adipocytes. A) – F) XF96 extracellular flux measurements of primary cultures of fully differentiated 129Sv/S1 brown UCP1-knockout (UCP1KO) adipocytes. A) Cells were pre-treated with an HSL inhibitor (HSLi, 20 μM final), an ATGL inhibitor (ATGLi, 40 μM final), or both inhibitors (ATGLi + HSLi) for 1 h prior to the measurement and inhibitors were present in the assay medium throughout the measurement. Isoproterenol-induced increase in oxygen consumption rate (OCR) was calculated. n = 18–24 wells from three independent biological experiments. One-way ANOVA followed by Tukey's HSD was applied. “a” indicates a significant difference from the control group, “b” from the HSLi group, and “c” from the ATGLi group. Not statistically significant (n.s.). B) Oligomycin delivered *via* the second injection (port “B”) was replaced with a combination of the ATGL and the HSL inhibitor. Portion of stimulated OCR sensitive to oligomycin or lipase-inhibitors was calculated. n = 21 wells from three independent biological experiments. A two-tailed t-test was applied. Not statistically significant (n.s.). C) Cells were pre-treated with a long-chain acyl-CoA synthetase inhibitor (Triacsin C, 5 μM final) for 1 h prior to the measurement and triacsin C was present in the assay medium throughout the measurement. Isoproterenol-induced increase in oxygen consumption rate (OCR) was calculated. n = 15–22 wells from three independent biological experiments. A two-tailed t-test was applied. Asterisk (∗) indicates a significant difference between the two groups. D) Cells were pre-treated with a DGAT1 inhibitor (DGAT1i, 40 μM final), a DGAT2 inhibitor (DGAT2i, 40 μM final), or both inhibitors (DGAT1i + DGAT2i) for 16 h prior to the measurement and inhibitors were present in the assay medium throughout the measurement. Isoproterenol-induced increase in oxygen consumption rate (OCR) was calculated. n = 20–21 wells from three independent biological experiments. One-way ANOVA followed by Tukey's HSD was applied. “a” indicates a significant difference from the control group, “b” from the DGAT2i group, and “c” from the DGAT1i group. Not statistically significant (n.s.). E) Dgat1 was knocked down in brown UCP1KO adipocytes with DsiRNA as described in “Material & Methods” and the isoproterenol-induced increase in OCR was calculated. n = 27–30 wells from three independent biological experiments. A two-tailed t-test was applied. Asterisk (∗) indicates a significant difference between the two groups. F) Palmitate conjugated to bovine serum albumin (Palmitate:BSA, 160 μM Palmitate:28 μM BSA final) or just BSA (28 μM final) was added to the BSA-free assay medium immediately prior to starting the measurement and ATP-linked OCR was quantified. n = 37–43 wells from three independent biological experiments. A two-tailed t-test was applied. Asterisk (∗) indicates a significant difference between the two groups. G) 129Sv/S1 brown wild type (WT) and UCP1KO cells were pre-treated as described in “Material & Methods”, glycerol and free fatty acids (FFAs) in the medium were quantified, and the amount of re-esterified FFA was calculated. 2-deoxyglucose (2DG, 50 mM final). n = two independent biological experiments (within each experiment, the content of 8 wells of each treatment level was pooled). Kruskal–Wallis two-way ANOVA followed by multiple comparisons with a Bonferroni-corrected Mann–Whitney U test was applied. Asterisk (∗) indicates a significant difference from the control group of the respective genotype. Number sign (#) indicates a significant difference between the two genotypes within one treatment level.

**Figure 4:**
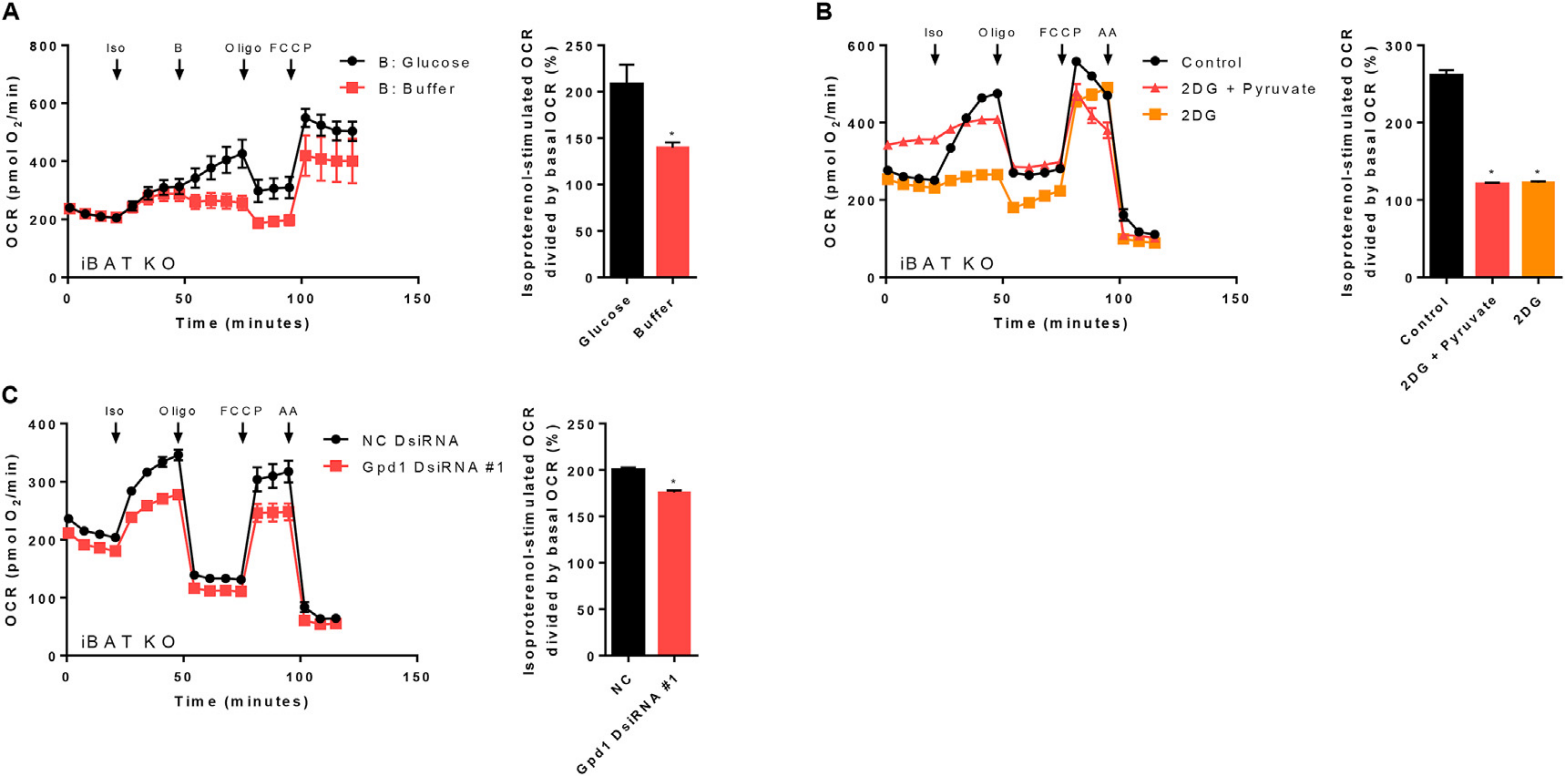
Glycolysis fuels UCP1-independent thermogenesis in brown adipocytes lacking UCP1. A) – C) XF96 extracellular flux measurements of primary cultures of fully differentiated 129Sv/S1 brown UCP1-knockout (UCP1KO) adipocytes. A) Cells were assayed in glucose-free medium. Glucose (25 mM final) or buffer was delivered *via* the second injection (port “B”) and the isoproterenol-induced increase in OCR was calculated. n = 10–16 wells from two independent biological experiments. A two-tailed t-test was applied. Asterisk (∗) indicates a significant difference between the two groups. B) Cells were pre-treated with 2-deoxyglucose (2DG, 50 mM final) or a combination of 2DG and pyruvate (2DG + Pyruvate; 2DG 50 mM final, pyruvate 5 mM final). Isoproterenol-induced increase in OCR was calculated. n = 16–28 wells from three independent biological experiments. One-way ANOVA followed by Tukey's HSD was applied. Asterisk (∗) indicates a significant difference from the control group. C) Gpd1 was knocked down in brown UCP1KO adipocytes with DsiRNA as described in “Material & Methods” and the isoproterenol-induced increase in OCR was calculated. n = 28–31 wells from three independent biological experiments. A two-tailed t-test was applied. Asterisk (∗) indicates a significant difference between the two groups.

Figure 2, Figure 3, Figure 4C, and Supplementary Figs. 2B and 2C:

$$\text{Isoproterenol}-\text{stimulated OCR divided by basal OCR }\left(\%\right)=\frac{\left(maximum\;after\;isoproterenol-minimum\;after\;antimycin\;A\right)}{\left(minimum\;prior\;to\;isoproterenol-minimum\;after\;antimycin\;A\right)}*100$$

Figure 2C:

$$\text{Isoproterenol}-\text{stimulated increase over basal OCR}\;\left(pmol\;O_{2}/min\right)=maximum\;after\;isoproterenol-minimum\;prior\;to\;injection\;"A"$$

Isoproterenol−stimulated increase over OCR after injection "A"=maximumafterisoproterenol−mimimumafterinjection"A"

Figure 3B:

$$\text{OCR sensitive to Oligomycin or lipase}-\text{inhibitors}\;\left(\%\right)=\;\frac{\left(maximum\;after\;isoproterenol-minimum\;after\;oligomycin/lipase-inhibitors\right)}{\left(maximum\;after\;isoproterenol-minimum\;prior\;to\;isoproterenol\right)}*100$$

Figure 3F:

$$\text{ATP}-\text{linked respiration}\;\left(pmol\;O_{2}/min\right)=mimimum\;prior\;to\;oligomycin-minimum\;after\;oligomycin$$

Figure 4A:

$$\text{Isoproterenol}-\text{stimulated OCR divided by basal OCR}\;\left(\%\right)=\frac{maximum\;after\;injection\;"B"}{minimum\;prior\;to\;isoproterenol}*100$$

### 2.7 Quantification of glycerol and free FAs in cell culture supernatant

Adipocytes were cultured in 96-well plates and the release of glycerol and free fatty acids into the medium was measured on day 8. Cells were washed twice with respiration base medium and respiration assay medium was added to a final volume of 180 μl per well (inhibitors were added to the assay medium at this point and were present in the medium during the measurement, see 2.8). The cell culture plate was incubated for 1 h at 37 °C in a laboratory non-CO_2_ incubator. After that, isoproterenol, oligomycin, or a combination of both was added to the medium. The cell culture plate was placed back into a laboratory non-CO_2_ incubator and incubated at 37 °C. After an additional hour, conditioned media were harvested and stored for further analysis. Free glycerol was determined with the Free Glycerol Determination Kit (Sigma–Aldrich) and free FAs were quantified with the NEFA-HR(2) Assay (FUJIFILM Wako Chemicals). Free FA re-esterification rate was calculated as previously described [10,30]. Free FAs re-esterified = 3 ∗ glycerol – free FAs.

### 2.8 Chemicals and inhibitors

**Table:** 

Action/inhibitor target	Chemical/inhibitor name	Final concentration in well (μM)	Incubation time (h)
Adrenergic-β receptor agonist.	Isoproterenol	0.1	acute
Inhibits adipose triglyceride lipase (ATGL). Impairs lipolysis.	Atglistatin	40	1
Inhibits hormone-sensitive lipase (HSL). Impairs lipolysis.	Hi-76-0079 [41]	20	1
Inhibits carnitine palmitoyltransferase I (CPTI). Impairs import of FAs into mitochondria.	Etomoxir	50	1
Impairs opening of the mitochondrial permeability transition pore.	Cyclosporin A	4.2	72
Competitive inhibition of diacylglycerol O-Acyltransferase 1 (DGAT1). Impairs TG synthesis and re-esterification of FAs.	T863	40	16
Inhibits diacylglycerol O-Acyltransferase 2 (DGAT2). Impairs TG synthesis.	PF-06424439	40	16
Inhibits sarco/endoplasmic reticulum Ca^2+^-ATPase (SERCA). Impairs uptake of Ca^2+^ into ER.	Thapsigargin	5	1
Selective Ca^2+^ chelator. Lowers intracellular Ca^2+^ levels.	BAPTA-AM	20	1
Shuts down glycolysis/competitive inhibition of glucose-6-phosphate isomerase.	2-deoxy-d-glucose (2DG)	50,000	1
Competitive inhibition of long chain fatty acyl-CoA ligase (or synthetase). Impairs activation of FAs.	Triacsin C	5	1

### 2.9 RT-qPCR

#### 2.9.1 Cultured cells

Total RNA was isolated by TRIsure™-chloroform extraction and subsequent column purification using columns from the SV Total RNA Isolation System (Promega) according to the manufacturer's recommendations. First-strand cDNA synthesis was performed with SensiFAST™ cDNA Synthesis Kit (Bioline). 5 ng of cDNA were employed per qPCR reaction. RT-qPCR was carried out on a LightCycler®480 Real-Time PCR System (Roche) using SensiMix™ SYBR® No-ROX Kit (Bioline). Relative transcript abundance was quantified with an arbitrary standard curve consisting of pooled cDNA from various samples. Relative transcript abundance was normalized to the expression of Gtf2b. Gene abbreviations and an overview of primer sequences are given in Table 1.

**Table 1:** Primer cell culture.

*Gene Name*	Gene ID	Forward primer	Reverse primer
*Atp2a2*	11938	ACCTTTGCCGCTCATTTTCCAG	AGGCTGCACACACTCTTTACC
*Dgat1*	13350	GGAATATCCCCGTGCACAA	CATTTGCTGCTGCCATGTC
*Dgat2*	67800	CCGCAAAGGCTTTGTGAA	GGAATAAGTGGGAACCAGATCAG
*Gpd1*	14555	CCTTGTGGACAAGTTCCCCTT	GACAGTCCTGATGACGGGTG
*Gtf2b*	229906	TGGAGATTTGTCCACCATGA	GAATTGCCAAACTCATCAAAACT

#### 2.9.2 Tissues

Total RNA was isolated and gene expression was evaluated as described [42]. Data were normalized to the geometric mean signal of two reference genes in iBAT (18Srna and Eef1a1) or to Eef1a1 in eWAT. For data normalization Ln, Log10, or sqrt transformation was used when needed. The delta–delta CT method [43] was used for calculating relative changes in gene expression between both tissues. Gene abbreviations and an overview of primer sequences are given in Table 2.

**Table 2:** Primer tissues.

Gene Name	Gene ID	Forward primer	Reverse primer
*18Srna/Rn18s*	19791	GCCCGAGCCGCCTGGATAC	CCGGCGGGTCATGGGAATAAC
*Acly*	104112	GTGGCGGGGAAGTGCTGTTTGA	TGTGCTCGGGCTGGGAAGGAC
*Aqp7*	11832	CTTGGGTTTTGGATTCGGAGTGAC	GGTCTCCGCCTGCAAAGTGGTTA
*Atgl/Pnpla2*	66853	CAAGGGGTGCGCTCTGTGGATGG	AGGCGGTAGAGATTGCGAAGGTTG
*Cd36*	12491	TGATACTATGCCCGCCTCTCC	TTCCCACACTCCTTTCTCCTCTAC
*Cidea*	12683	ACTGGCCTGGTTACGCTGGTG	GGCTATTCCCGATTTCTTTGGTTG
*Dgat1*	13350	TGGCCAGGACAGGAGTATTTTTGA	CTCGGGCATCGTAGTTGAGCA
*Dgat2*	67800	TGCCCTACTCCAAGCCCATCACC	TCAGTTCACCTCCAGCACCTCAGTCTC
*Eef1a1*	13627	TGACAGCAAAAACGACCCACCAAT	GGGCCATCTTCCAGCTTCTTACCA
*Fas/Fasn*	14104	TGGGTGTGGAAGTTCGTCAG	GTCGTGTCAGTAGCCGAGTC
*Gk*	14933	TCGTTCCAGCATTTTCAGGGTTAT	TCAGGCATGGAGGGTTTCACTACT
*Gpd1*	14555	GCAGACACCCAACTTTCGCATCA	CCGCCGCCTTGGTGTTGTCA
*Lcad/Acad*l	11363	TGGCATCAACATCGCAGAGAAACA	ACCGATACACTTGCCCGCCGTCAT
*Ldha*	16828	GTTGCCGGCGTCTCCCTGAA	CGTAGGCACTGTCCACCACCTG
*Ldhb*	16832	TCCAGTGTGGCTGTGTGGAGCG	AGGCCGATGGCCCAGTTGGT
*Lpl*	16956	AGCCCCCAGTCGCCTTTCTCCT	TGCTTTGCTGGGGTTTTCTTCATTCA
*Mct1/Slc16a1*	20501	GGCCACCACTTTTAGGCCGC	TCGTCGACATCGGTGCTGGC
*Mct4/Slc16a3*	80879	GGGCAGTGGTCTGTTCACCCT	ATGGGGCTCCCTCCCTTGCTA
*Pc*	18563	CCCCTGGATAGCCTTAATACTCGT	TGGCCCTTCACATCCTTCAAA
*Pepck/Pck1*	18534	GGCAGCATGGGGTGTTTGTAGGA	TTTGCCGAAGTTGTAGCCGAAGAAG
*Pygl*	110095	AGCCAGCGCCTCGGGGTAAT	ACGCGGTGAACGGTGTAGCA
*Ucp1*	22227	CACGGGGACCTACAATGCTTACAG	GGCCGTCGGTCCTTCCTT

**Table 3:** Energy balance, tissue weights, and blood parameters of C57BL/6J wild type (WT) and UCP1-knockout (UCP1KO) mice acclimated to different ambient temperatures: 30 °C “warm-acclimated” (WA), 20 °C “mild cold-acclimated” (MCA), or 6 °C “cold-acclimated” (CA), for at least three weeks. n = 9–10 animals from two independent experiments. A two-way ANOVA followed by Tukey's HSD was applied. “a” indicates a significant difference from the WA group of the respective genotype. “b” indicates a significant difference from the MCA group of the respective genotype. “c” indicates a significant difference between the two genotypes within one treatment level. Abbreviations: AT, adipose tissue; eWAT, epididymal white adipose tissue; iBAT, interscapular brown adipose tissue; NEFA, non-esterified fatty acid; iWAT, inguinal white adipose tissue; TG, triglyceride.

	WA		MCA		CA	
	*WT*	*UCP1KO*	*WT*	*UCP1KO*	*WT*	*UCP1KO*
Food consumption (g day ^−1^)	2.70 ± 0.10	3.0 ± 0.1	4.4 ± 0.1^a^	4.8 ± 0.1^a^	7.4 ± 0.1^a,b^	7.9 ± 0.2^a,b^
Body weight final (g)	27.51 ± 0.66	28.47 ± 1.15	26.97 ± 0.55	28.05 ± 0.41	27.69 ± 0.45	27.63 ± 0.36
*Weight of AT depots*						
eWAT (mg)	500 ± 48	497 ± 84	324 ± 21^a^	358 ± 31	194 ± 16^a,b^	211 ± 11^a,b^
iWAT (mg)	273 ± 21	280 ± 35	213 ± 14	222 ± 12	167 ± 14^a,b^	237 ± 13^c^
iBAT (mg)	117 ± 7	108 ± 8	94±4^a^	185 ± 13^a,c^	118±6^b^	157 ± 16^a,c^
*Liver*						
Weight (mg)	1269 ± 51	1376 ± 82	1430 ± 40	1676 ± 47^a,c^	1531 ± 68^a^	1683 ± 65^a^
TG (mg per g tissue)	16.09 ± 1.73	16.78 ± 1.12	14.91 ± 1.63	22.91 ± 4.19	13.22 ± 1.46	18.57 ± 1.80
*Quadriceps muscle*						
Weight (mg)	270 ± 8	269 ± 10	264 ± 8	296±8^a,c^	245±5^a^	235±6^a,b^
TG (mg per g tissue)	8.03 ± 1.53	10.72 ± 3.25	6.78 ± 1.61	11.86 ± 4.28	5.37 ± 0.79	10.75 ± 2.44
*Heart*						
Weight (mg)	124 ± 3	138±8^c^	137±3^a^	164±5^a,c^	181±7^a,b^	222±9^a,b,c^
*Plasma levels*						
TG (mmol l ^−1^)	1.13 ± 0.07	1.03 ± 0.10	1.08 ± 0.06	0.47 ± 0.04^a,c^	0.80 ± 0.09^a,b^	0.37 ± 0.04^a,c^
NEFA (mmol l ^−1^)	0.33 ± 0.04	0.31 ± 0.03	0.33 ± 0.04	0.27 ± 0.03	0.25 ± 0.03	0.13 ± 0.02^a,b,c^
Cholesterol (mmol l − 1)	2.03 ± 0.14	1.89 ± 0.12	1.75 ± 0.05^a^	1.44 ± 0.05^a,c^	1.16 ± 0.11^a,b^	1.01 ± 0.08^a,c^
Glucose (mg dl ^−1^)	7.8 ± 0.4	7.2 ± 0.3	7.8 ± 0.3	7.8 ± 0.4	7.9 ± 0.5	8.4 ± 1.0
Insulin (ng ml ^−1^)	0.78 ± 0.17	1.09 ± 0.33	0.75 ± 0.14	0.64 ± 0.18	0.73 ± 0.16	1.85 ± 0.28^a,b,c^

### 2.10 Western blotting

Frozen iBAT samples (−80 °C) were weighed and RIPA buffer (150 mM NaCl, 1% Nonidet NP-40, 1% sodium deoxycholate, 0.1% SDS, 50 mM Tris pH 8.0, 1 mM PMSF, 1 μg/ml leupeptin, 1 μg/ml aprotinin, 1 μg/ml pepstatin) was added in 10x dilution (approximately tissue:buffer 1:9). Samples were homogenized in ice-cold buffer using a ball mill MM40 (Retsch, Germany; 30 s^−1^, 3 min). Protein concentration was assessed by bicinchoninic acid method, and the amount of protein per whole tissue depot was calculated. iBAT homogenates (10 μg protein/well) were loaded onto a 10% Tricine SDS-PAGE gel. Electrophoresis and western blotting were performed as described before [44]. OXPHOS proteins were detected by using Total OXPHOS Blue Native WB Antibody Cocktail (Abcam, ab110412, 1:250), which includes antibodies against following OXPHOS subunits: NDUFA9 (complex I), SDHA (complex II), UQCRC2 (complex III), COX4 (complex IV), ATP5A1 (complex V). Alexa Fluor 680 goat anti-mouse was used as secondary antibody. GAPDH was detected as reference protein; GAPDH (14C10) Rabbit mAb (#2118, Cell Signaling, 1:1000). IR Dye 800 CW Donkey anti-Rabbit IgG Secondary Antibody (Li-cor) was used as secondary antibody. Fluorescence was detected using an Odyssey Imager (Li-cor) and the signal was quantified with Image Lab software (Bio-Rad Laboratories). Intensities of OXPHOS proteins were normalized to GAPDH and recalculated per whole iBAT depot.

### 2.11 Proteome analysis

#### 2.11.1 Cells lysis and protein digestion

Cells lysis was performed by adding 100 μl of lysis buffer consisting of 8 M urea in 50 mM Tris–HCl pH 8, in the presence of EDTA-free protease inhibitors cocktail (Roche) and phosphatase inhibitors mixture. Lysates were then sonicated in a Bioruptor Pico (Diagenode) using a 10 cycles program (30 s ON, 30 s OFF), and cleared by centrifugation for 10 min at 20,000 g and 4 °C. iBAT samples were further lysed by mechanical disruption with single use pestles in protein Lobind tubes and on ice. Protein lysates (100–200 μg) were reduced with 10 mM DTT at 37 °C for 40 min, and alkylated with 55 mM chloroacetamide at room temperature for 30 min in the dark. For tryptic digestion, proteins were digested overnight at 37 °C with sequencing grade modified trypsin (Promega, 1:50 enzyme-to-substrate ratio) after 4-fold dilution with 50 mM Tris–HCl, pH 8. Digests were acidified by addition of formic acid (FAc) to 5% (v/v) and desalted using Sep-Pak C18 cartridges, as previously described [45]. Eluted peptides were then frozen at −80 °C and dried in vacuo.

#### 2.11.2 TMT labeling and peptide fractionation

TMT 10-plex or TMTpro 16-plex labeling was performed as previously described [45]. In brief, each digest was resuspended in 20 μL of 50 mM HEPES (pH 8.5). Five μL of 11.6 mM TMT reagents stock solution (Thermo Fisher) in 100% anhydrous ACN were then added to each sample. Labeling reaction was carried for 1 h at 25 °C, and quenched by adding 2 μL of 5% hydroxylamine. Peptide solutions were pooled and acidified using 20 μL of 10% FAc. The pooled sample was dried in vacuo, desalted and stored dried at −80 °C until further use.

For whole proteome analysis of WT and UCP1KO, dried peptides were re-suspended in 10 mM ammonium acetate, pH 4.7, and subjected to trimodal mixed mode chromatography on an Acclaim Trinity P1 2.1 × 150 mm, 3 μm column (Thermo Fisher) operated by a Dionex Ultra 3000 HPLC system (Thermo Fisher) [46]. A total of 32 fractions were collected.

For whole proteome analysis of iBAT, dried peptides were re-suspended in 2.5 mM ammonium bicarbonate, pH 8 and subjected to high pH RP fractionation instead, using a Waters XBridge BEH130C18 2.1 × 150 mm, 3.5um column (Waters). Buffer A was 25 mM ammonium bicarbonate (pH = 8), buffer C was 100% ultrapure water, buffer D was 100% CAN. The proportion of buffer A was kept at 10% at all times. Peptides were separated by a linear gradient from 7% D to 45% D in 44 min, and followed by a linear gradient from 45% D to 85% D in 6 min. A total of 96 fractions were collected every 30 s, and then concatenated to 48 fractions by adding fraction 49 to fraction 1, fraction 50 to fraction 2 and so forth.

All fractions were dried in vacuo and stored at −20 °C until nLC-MS/MS analysis.

#### 2.11.3 LC-MS/MS

Nano flow LC-ESI-MS measurements were performed using a Dionex Ultimate 3000 UHPLC + system coupled to a Fusion Lumos Tribrid mass spectrometer (Thermo Fisher). Peptides were delivered to a trap column (75 μm × 2 cm, packed in-house with 5 μm Reprosil C18 resin; Dr. Maisch) and washed using 0.1% FAc at a flow rate of 5 μL/min for 10 min. Subsequently, peptides were transferred to an analytical column (75 μm × 45 cm, packed in-house with 3 μm Reprosil C18 resin, Dr. Maisch) applying a flow rate of 300 nL/min. Peptides were chromatographically separated using a 50 min linear gradient from 8% to 34% solvent B (0.1% FAc, 5% DMSO in ACN) in solvent A (0.1% FAc in 5% DMSO).

For the iBAT samples, the Fusion Lumos Tribrid mass spectrometer was coupled to a micro-flow LC-MS/MS system using a modified Vanquish pump (Thermo Fisher). Chromatographic separation was performed *via* direct injection on a 15 cm Acclaim PepMap 100C18 column (2 μm, 1 mm ID, Thermo Fisher Scientific) at a flow rate of 50 μl/min, using a 25 min linear gradient (4%–28%) of solvent B (0.1% FAc, 3% DMSO in ACN) and solvent A (0.1% FAc in 3% DMSO). The total measurement time for each sample was 27 min.

The Fusion Lumos was operated in a data-dependent acquisition (DDA) to automatically switch between MS and MS/MS. Briefly, survey full-scan MS spectra were recorded in the Orbitrap from m/*z* 360 to 1300 at a resolution of 60K, using an automatic gain control (AGC) target value of 4e5 charges and maximum injection time (maxIT) of 50 ms.

For the MS3-based TMT method, initial MS2 spectra for peptide identification were recorded in the ion trap in rapid scan mode with a top speed approach using a 2-s duration (isolation window m/*z* 0.7, AGC target value of 1e4, maxIT of 35 ms). Fragmentation was set to CID, with a NCE of 35% and activation Q of 0.25. Then, for each peptide precursor, an additional MS3 spectrum for TMT quantification was obtained in the Orbitrap at 50K resolution (AGC of 5e4 charges, maxIT of 86 ms). The precursor was fragmented as for the MS2 analysis, followed by synchronous selection of the 10 most intense peptide fragments and further fragmentation *via* HCD using a NCE of 55%. Dynamic exclusion was set to 90 s.

For the analysis of the TMTpro 16-plex samples, the following parameters were modified: top speed method duration of 1.2-s, isolation window m/*z* 0.6, AGC target value of 1.2e4, maxIT of 40 ms, fragmentation was set to HCD with a NCE of 32%. MS3 ACG was set 1e5 charges, number of notches 8, and dynamic exclusion was set to 50 s.

#### 2.11.4 Data processing

Peptide and protein identification and quantification was performed using MaxQuant (version 1.6.0.43) with its built in search engine Andromeda [47]. Spectra were searched against the UniProtKB database (Mus musculus, UP000000589, 55431 entries downloaded on 12.2019). Enzyme specificity was set to trypsin, allowing for 2 missed cleavages, and the search included cysteine carbamidomethylation as a fixed modification and Ntem-acetylation of protein, oxidation of methionine as variable modifications. TMT10 was set as label within a reporter ion MS3 experiment type. Precursor tolerance was set to 5 ppm, and fragment ion tolerance to 20 ppm. Results were adjusted to 1% false discovery rate at protein, peptide, and site levels.

TMTpro 16-plex raw data file recorded by the mass spectrometer were processed and quantified with Proteome Discoverer (version 2.4, Thermo Scientific). Peak lists were generated with Proteome Discoverer with a signal-to-noise threshold of 1.5 and searched against the UP000000589 UniProtKB database using SequestHT as search engine. The database search was performed with the following parameters: a mass tolerance of ±10 ppm for precursor masses, ±0.6 Da for HCD-Ion trap fragment ions; two missed cleavages allowed; and cysteine carbamidomethylation as a fixed modification. Methionine oxidation and protein N-term acetylation were set as variable modifications. The enzyme was specified as trypsin, with a minimum peptide length of 6 amino acids. All PSMs were validated with Percolator [48], and results were adjusted to 1% false discovery rate at protein, peptide and PSM level within Proteome Discoverer. TMTpro was set as label (static modification) and used by the Reporter Ions Quantifier node for the peptide and protein quantification. Default settings were used.

The mass spectrometry proteomics data have been deposited in the ProteomeXchange Consortium *via* the PRIDE partner repository [49] with the dataset identifier PXD025854.

#### 2.11.5 Bioinformatic analysis

Data analysis was performed in Perseus (v. 1.6.1.1 and v. 1.6.15.0) [50] and R [51]. Proteome datasets were filtered to remove contaminants and decoy identifications, before performing data normalization of the intensity values by median centering, as implemented in Perseus.

For statistical tests, only proteins that have been quantified in at least 3 biological replica were considered. To identify significantly regulated proteins Welch's t-test was used, corrected for multiple hypotheses using a Benjamini-Hochberg false discovery rate of 5%.

iBAT proteome dataset was filtered to retain only proteins that have been quantified in all the 4 biological replica in at least one experimental condition, and missing values were imputed in Perseus using default settings. Here, to identify significantly modulated proteins ANOVA test was used, corrected for multiple hypotheses using a Benjamini-Hochberg false discovery rate of 5%, and followed by Tukey HSD post-hoc test in R. Heatmaps were generated in R using the package “pheatmap”. Normalized protein intensities were scaled in the row direction and rows were clustered by Euclidean distance.

Gene ontology (GO) annotations were downloaded from UniProt. Categorical annotation was supplied by Gene Ontology biological process, molecular function, and cellular component; and the KEGG pathway database. The GO terms enrichment was calculated on the basis of a fisher's exact test with a false discovery rate value of 0.05. Scores were plotted as a two-dimensional annotation enrichment score [52].

### 2.12 Electron microscopy

For electron microscopy, cells were grown in flat BEEM capsules (Plano GmbH, Wetzlar, Germany). Samples were fixed with 2.5% glutaraldehyde in 0.1 M sodium cacodylate buffer, pH 7.4 (Electron Microscopy Sciences, Hatfield, USA) for 24 h at minimum. Thereafter glutaraldehyde was removed and samples were washed three times with 0.1 M sodium cacodylate buffer, pH 7.4. Postfixation and prestaining was done for 30 min with 1% osmium tetroxide (10 ml 4% osmium tetroxide (Electron Microscopy Sciences), 10 ml ddH2O, 10 ml 3.4% sodium chloride and 10 ml 4.46% potassium dichromate (pH adjusted to 7.2 with KOH (Sigma Aldrich)). Samples were washed three times with 0.1 M sodium cacodylate buffer, pH 7.4 and dehydrated with an ascending ethanol series (10 min with 30%, 50%, 70%, 90% and 96%, respectively, two times 20 min with 100%, and 20min with dehydrated EtOH (molecular sieve 3A°)). For embedding, EtOH was stepwise replaced by Epon (3.61 M glycidether 100, (Serva Electrophoresis GmbH), 1.83 M methylnadicanhydride (Serva Electrophoresis GmbH), 0.92 M dodecenylsuccinic anhydride (Serva Electrophoresis GmbH), 5.53 mM 2,4,6-Tris(dimethylaminomethyl)phenol (Serva Electrophoresis GmbH)). (1) 20min with Epon:100% EtOH at a ratio of 1:1, (2) 20 min with Epon:100% EtOH at a ratio of 2:1, (3) Epon overnight, (4) replacement with fresh Epon. The embedded samples were hardened at 60 °C for 48 h. Ultrathin sections were sliced with an Ultramicrotome (Ultracut E; Reichert und Jung, Germany) and automatically stained with UranyLess EM Stain (Electron Microscopy Sciences) and 3% of lead citrate (Leica, Wetzlar, Germany) using the contrasting system Leica EM AC20 (Leica, Wetzlar, Germany). The samples were examined with a JEOL-1200 EXII transmission electron microscope (JEOL GmbH, Freising, Germany). Images were taken using a digital camera (KeenViewII, Olympus, Germany) and processed with the iTEM software package (anlySISFive; Olympus, Germany). Images were analyzed with Fiji [53]. Cytoplasmic and peridroplet mitochondria were manually counted. Mitochondria were classified as cytoplasmic mitochondria, if no clear contact site between mitochondrial and lipid droplet membrane was identified. Mitochondria were classified as peridroplet mitochondria, if at least one contact site was detected (the mitochondrial membrane could not be distinguished from the lipid droplet membrane). Lipid droplet perimeter as well as the sections of the lipid droplet perimeter occupied by mitochondria was manually quantified.

### 2.13 Statistical analyses

If not stated otherwise, data are presented as mean ± SEM.

Comparison of two groups: Normal distribution of data was tested using the Shapiro–Wilk test. Normally distributed data were compared with an unpaired t-test. When data violated the assumptions for a t-test, a Welch-test was performed.

Comparison of three or more groups: Normal distribution of residuals was tested using the Shapiro–Wilk test, and homogeneity of variance was assessed by Levene's test. If ANOVA's assumptions were met, one-way or two-way ANOVA was performed followed by Tukey's HSD multiple comparisons. When data violated the assumptions for an ANOVA, a Kruskal–Wallis one-way or two-way ANOVA was performed followed by multiple comparisons with a Bonferroni-corrected Mann–Whitney U test.

Values of p < 0.05 were considered statistically significant.

## 3 Results

### 3.1 Adrenergic stimulation causes an acute upregulation of proteins related to Ca^2+^ and lipid metabolism in brown UCP1KO adipocytes

Several potentially thermogenic futile substrate cycles in adipose tissue of WT and UCP1KO mice have been reported [[23], [24], [25],^31^]. We hypothesized that isoproterenol treatment of brown adipocytes from UCP1KO mice would cause an immediate acceleration of futile substrate cycling, and entail a consecutive upregulation of the involved enzymes to recruit additional capacity. Therefore, we embarked on probing the cellular response of brown adipocytes of WT and UCP1KO mice to acute adrenergic stimulation at the protein level to identify differentially expressed candidates for further investigation in an unbiased manner (Figure 1A). Based on the assumption that futile substrate cycles contributing to NST are preferentially found in adipocytes lacking UCP1, we performed a two-dimensional pathway enrichment analysis [52] and focused primarily on the terms, which were downregulated or unchanged in WT cells, and upregulated in KO cells (Figure 1B). In a broad sense, terms meeting these criteria were either related to Ca^2+^ metabolism (“cardiac muscle contraction”, “muscle contraction”), lipid metabolism (“cellular lipid metabolic process”, “glycerophospholipid metabolism”), or the electron transport chain (“oxidative phosphorylation”, “hydrogen transport”). These genotype-dependent differences in the proteome upon adrenergic stimulation can be further assigned to specific cellular compartments. Significantly adrenergically regulated proteins of brown UCP1KO adipocytes were, based on their annotation, preferentially localized to the endoplasmic reticulum (ER), lipid droplets, and the inner mitochondrial membrane (Supp. Figure 1A). When looking at individual enzymes involved in the transport of Ca^2+^ into the ER (SERCA isoforms) and the efflux of Ca^2+^ back into the cytosol, we correspondingly observed a strong induction only in UCP1KO adipocytes (Supp. Figure 1B). Additionally, brown adipocytes lacking UCP1 showed a more pronounced upregulation of enzymes that directly or indirectly participate in TG synthesis compared to WT adipocytes (Supp. Figure 1C, left). On the contrary, we saw a marked reduction of proteins catalyzing the provision of acetyl-CoA, or DNL, as well as transcription factors that positively regulate the lipogenic machinery in UCP1KO cells, while we could not detect a significant change in WT adipocytes (Supp. Figure 1C, right).

Taken together, changes in the cellular proteome upon adrenergic stimulation that were particularly detected in brown UCP1KO adipocytes suggested the presence of a futile Ca^2+^ cycle and a futile TG substrate cycle acting separately or in a concerted manner.

### 3.2 The contribution of futile Ca^2+^ cycling and futile TG cycling to UCP1-independent thermogenesis

It has been known for a long time that adipocytes with very low UCP1 levels drastically increase their oxygen consumption in response to an adrenergic stimulus [54], while not too long ago the same phenomenon was described in brown and beige UCP1KO adipocytes [55]. The decisive difference between brown WT and UCP1KO adipocytes (i.e. *in vitro* differentiated adipocytes derived from iBAT of WT and UCP1KO mice) is that stimulated respiration rates in WT cells are largely driven by UCP1, and consequently oligomycin-insensitive. On the contrary, oxygen consumption in UCP1KO cells is not affected by cyclosporin A (Supp. Figure 2A), which effectively rules out the possibility that mitochondrial permeability transition pore opening explains this effect, and at the same time it is almost fully sensitive to oligomycin treatment proving that brown UCP1KO adipocytes markedly ramp up aerobic ATP production following the addition of isoproterenol (Figure 2A).

To decipher the mechanism underlying elevated oxygen consumption and ATP turnover of brown UCP1KO adipocytes we concentrated first on ATP-dependent Ca^2+^ cycling that was proposed to mediate NST in beige UCP1KO adipocytes [24]. Mechanistically, adrenergic signalling is thought to activate sarco/endoplasmic reticulum Ca^2+^ ATPase2b (SERCA2b) and the ryanodine receptor 2 (RyR2) leading to ATP-dependent uptake of Ca^2+^ into the ER by SERCA2b and the transport of Ca^2+^ back into the cytosol *via* RyR2. This increased Ca^2+^ flux uncouples mitochondrial oxygen consumption from oxidative phosphorylation. To test the contribution of futile Ca^2+^ cycling to NST in our model of brown UCP1KO adipocytes, we knocked down Atp2a2, which is coding for SERCA2b. Cells treated with a DsiRNA targeting Atp2a2 showed a small non-significant reduction in respiration rates following the addition of isoproterenol compared to the control group, despite having an 80% reduced (not shown) endogenous expression of Atp2a2 (Figure 2B). However, as another SERCA isoform might have compensated for the knockdown of Atp2a2, or a different isoform besides SERCA2b may mediate Ca^2+^ cycling in our experimental system, we treated UCP1KO adipocytes with thapsigargin, a potent non-competitive inhibitor of all SERCA isoforms. In line with the outcome of the knockdown experiments, pharmacological inhibition of SERCA did not decrease maximal oxygen consumption rates in response to adrenergic stimulation (Figure 2C, left). Even though the numerical increase induced by isoproterenol was significantly smaller in cells treated with thapsigargin, this is only an artefact, since the acute injection of thapsigargin already lead to higher respiration rates (Figure 2C, middle & right). To finally rule out any major contribution of futile Ca^2+^ cycling to cellular thermogenesis, we sought to interrupt intracellular Ca^2+^ flux in an untargeted manner by depleting endogenous Ca^2+^ levels using BAPTA, but failed to detect an effect on ATP turnover driving respiration (Figure 2C). Although the proteome analysis has revealed an upregulation of enzymes mediating a putative Ca^2+^ substrate cycle in brown UCP1KO adipocytes, we demonstrated that Ca^2+^ cycling is not the cause of increased energy turnover in UCP1 ablated adipocytes during adrenergic stimulation.

Accordingly, we continued to further pursue the exact molecular source of NST in UCP1KO adipocytes. The second candidate identified by our preceding analysis was a futile substrate cycle related to TG metabolism. In this case, we have chosen a top-bottom approach starting from very basic and general pathways, and then proceeding to refine subcomponents in yet greater detail. Lipolysis is a key pathway in lipid metabolism directly linked to and downstream of canonical adrenergic receptor-cAMP-PKA-signalling [56]. ATGL and HSL are the main lipases, which predominantly mediate the enzymatic hydrolysis of tri – and diacylglycerol into FAs and acylglycerols. Blocking HSL activity, ATGL activity, or combining both inhibitors reduced the isoproterenol-induced rise in oxygen consumption of brown UCP1KO adipocytes by around 25%, 60%, and 85%, respectively (Figure 3A). This proves that FAs released in response to an adrenergic stimulus are not inevitably required for mitochondrial ATP synthesis, but instead directly or indirectly participate in ATP-consuming reactions related to thermogenesis in the absence of UCP1.

Next, we tested whether a rapid surge in FA levels only provides the initial impulse or whether FAs need to be permanently released to keep the cycle going. Acute inhibition of ATGL and HSL activity during active lipolysis immediately diminished ATP-dependent oxygen consumption equivalently to oligomycin regarding kinetics and efficacy (Figure 3B). Consequently, the futile substrate cycle in brown UCP1KO adipocytes strictly depends on active lipolysis, because it is not only initiated, but also sustained by a continuous supply of FAs provided by ATGL and HSL lipase activity.

Of note, two mechanistically different futile cycles regarding lipid metabolism have been proposed in the past: A cycle comprising lipolysis and re-esterification of FAs [[10], [11], [12], [13], [14], [15], [16], [17], [18], [19], [20]], and a cycle consisting in degradation of FAs through beta-oxidation, and *de novo* lipogenesis [21]. Treating cells with etomoxir, an inhibitor of carnitine palmitoyltransferase 1 (CPT1) as the rate limiting enzyme of the carnitine shuttle, did not influence oxygen consumption rates of brown UCP1-knockout adipocytes upon adrenergic stimulation (Supp. Figure 2A). Thus, we could narrow down the subcellular localization, i.e. exclude the mitochondrial matrix as a compartment of interest, and rule out the second option “breakdown of FAs followed by FA re-synthesis”. Based on these findings and a very characteristic proteome signature, we favored the idea that murine brown UCP1KO adipocytes would immediately re-esterify a significant portion of FAs, which are released following adrenergic stimulation, onto glycerol-3-phosphate (G3P) or mono- and diglycerides. Since an adrenergic stimulus, such as isoproterenol, triggers lipolysis and TG synthesis at the same time [57,58], which are theoretically neutralizing each other's actions, one important criterion defining a futile substrate cycle is already met. The second prerequisite, dissipation of chemical energy as heat, would be fulfilled by the mandatory ATP-dependent activation of FAs preceding re-esterification onto a glycerol backbone. Therefore, we treated cells with triacsin C, a substance inhibiting long-chain acyl-CoA synthetase, and indeed, diminishing FA activation attenuated stimulated oxygen consumption rates by 50% (Figure 3C). This clearly indicates that less FAs were activated translating into a reduced ATP demand.

Based on these findings we inferred whether re-esterification of FAs is causally linked to the increase in oxygen consumption of UCP1KO adipocytes upon adrenergic stimulation. The final step in the synthesis of TGs and the quantitatively most important reaction in terms of (re-)esterification of FAs is catalyzed by diacylglycerol O-acyltransferase 1 (DGAT1) and DGAT2 [59]. Blocking the action of DGAT1 diminished the isoproterenol-induced increase in oxygen consumption rates by 50%, whereas DGAT2 inhibition did not significantly affect stimulated respiration (Figure 3D). Pharmacological inhibition of DGAT2 only slightly lowered basal oxygen consumption, which supports the notion that DGAT1 closely interacts with ATGL during lipolysis and FA re-esterification [60], while DGAT2 catalyzes *de novo* synthesis of TGs under normal conditions [61]. In a parallel approach, we depleted DGAT1 using siRNA technology to confirm its role in re-esterification during lipolysis. Cells that have received a DsiRNA targeting DGAT1 had 40% reduced oxygen consumption rates following adrenergic stimulation clearly indicating less futile ATP dependent re-esterification of FAs in brown UCP1KO adipocytes (Figure 3E). Moreover, the combined inhibition of DGAT1 and DGAT2 lowered oxygen consumption after the addition of isoproterenol even further by up to 70% (Figure 3D). This finding probably reflects the fact that DGAT2 can compensate the loss of DGAT1 activity but only to some very limited extent. Additionally, it should be noted that a bolus of exogenously supplied FAs without adrenergic stimulation was in fact sufficient to activate ATP dependent re-esterification in UCP1KO cells (Figure 3F). This finding is of particular importance, as it provides a completely new perspective on futile lipid cycling, which may not be an exclusively intracellular cycle, but could represent an inter-organ cycle involving different adipose tissue depots or even non-adipose tissue organs, such as liver or skeletal muscle [19].

Finally, to obtain an additional and more direct readout of lipid cycling activity, we calculated re-esterification rates based on the release of FAs and glycerol of cultured adipocytes under various conditions [10,30]. Although this method does not consider recycling of glycerol by glycerol kinase, and that a small amount of FAs is metabolized through beta-oxidation and used for phospholipid synthesis, it provides an adequate approximation. In the basal state brown WT and UCP1KO adipocytes re-esterified a comparable amount of FAs (Figure 3G). UCP1KO adipocytes massively increased their re-esterification rate upon adrenergic stimulation, whereas WT adipocytes only showed a small non-significant response. In good agreement with our findings based on respirometry, inhibiting mitochondrial ATP synthesis did hardly affect the stimulated FA cycling rate of WT adipocytes, while 50% of the cycling rate in brown UCP1KO adipocytes were sensitive to oligomycin treatment (Figure 3G). Blocking lipolysis completely prevented any increase in re-esterification rates independent of the genotype. Reducing ATP-dependent activation of FAs prior to DGAT1 mediated re-esterification attenuated re-esterification rates by 65% in UCP1KO cells, whereas WT adipocytes, again, were not significantly affected. Lastly, the addition of 2-deoxyglucose (2DG), a glucose analogue that is blocking glycolysis at the level of hexokinase and glucose-6-phosphate isomerase, caused a massive reduction in the amount of re-esterified FAs, but only in UCP1KO cells.

Taken together, we demonstrate that brown adipocytes recruit a futile cycle of lipolysis and FA re-esterification to generate heat in the absence of UCP1. Our findings prove that activating the adrenergic receptor-cAMP-PKA-signalling cascade causes lipid turnover to immediately increase [62]. The main steps defining this futile substrate cycle include ATGL mediated hydrolysis of TGs, activation of FAs by long-chain acyl-CoA synthetase under the consumption of ATP, and esterification of FAs onto diglycerides catalyzed by DGAT1.

### 3.3 A link between glucose metabolism and futile TG cycling

Interestingly, not only the proteome analysis revealed “glycolysis/gluconeogenesis” as significantly regulated in response to an adrenergic stimulus (Supp. Figure 1D), but we also found a surprisingly strong and causal link between glucose metabolism, i.e. treatment with 2DG, and the FA cycling rate in UCP1 ablated adipocytes (Figure 3G). This observation is particularly noteworthy, as glucose uptake per se into brown adipose tissue and cultured brown adipocytes functions independent of UCP1 [30,63,64]. Nevertheless, the exact intracellular metabolic fate of glucose in brown UCP1KO adipocytes remains unclear. Glucose can (i) be metabolized to pyruvate and feed into the TCA cycle, (ii) be converted to lactate *via* anaerobic glycolysis supporting ATP synthesis, and (iii) act as a building block for various metabolic intermediates, such as G3P [65]. Based on our previous findings, we hypothesized that G3P derived from glycolysis may represent a significant source of glycerol backbones onto which activated FAs can be attached. When brown UCP1KO adipocytes were stimulated with isoproterenol in the absence of glucose, the increase in oxygen consumption was massively blunted compared to cells that were assayed in the presence of glucose (Figure 4A), whereas glucose removal had no effect in WT cells (Supp. Figure 2B). However, as soon as glucose was re-introduced into the system, oxygen consumption of UCP1KO adipocytes immediately started to rise. To finally prove that a considerable amount of glucose is converted to G3P in brown UCP1KO adipocytes, cells were treated with 2DG, which is blocking glycolysis. Yet again, the surge in oxygen consumption following adrenergic stimulation was genotype-dependent and almost completely absent in UCP1KO cells, i.e. reduced by 85% compared to the control (Figure 4B), while the effect of 2DG was by far not as pronounced in WT adipocytes (Supp. Figure 2C). However, bypassing glycolysis with the addition of exogenous pyruvate was not able to adequately restore respiration rates, which indicates that brown UCP1KO adipocytes do not primarily require glucose to produce pyruvate but possibly to generate G3P backbones for FA re-esterification. Cellular G3P levels are tightly controlled by the G3P shuttle, which consists of the two enzymes glycerol-3-phosphate dehydrogenase 1 (GPD1) and GPD2 [66]. The cytosolic isoform GPD1 catalyzes the conversion of dihydroxyacetonephosphate to G3P. Consequently, if a significant portion of glucose was converted to G3P and GPD1 activity contributed to the provision of G3P backbones, lowering the expression of GPD1 would negatively affect the capacity to re-esterify FAs without compromising oxidative capacity. In line with our hypothesis, DsiRNA mediated reduction of Gpd1 expression by 70% (not shown) resulted in an approximately 30% decrease of oxygen consumption rates after brown UCP1KO adipocytes were stimulated with isoproterenol (Figure 4C).

These findings highlight the interdependence of glucose and lipid metabolism, and clearly suggest that brown UCP1KO adipocytes do not exclusively convert glucose to pyruvate, but rather metabolize glucose to G3P *via* dihydroxyacetonephosphate. Thus, besides transporting reducing equivalents, G3P can serve as a backbone for re-esterification of FAs and TG synthesis sustaining futile lipid cycling upon adrenergic stimulation. This dual role could partially explain the impressive glucose uptake rates of activated BAT in UCP1KO mice.

### 3.4 Form follows function: the association of lipid droplets and mitochondria in brown adipocytes

**Figure 5:**
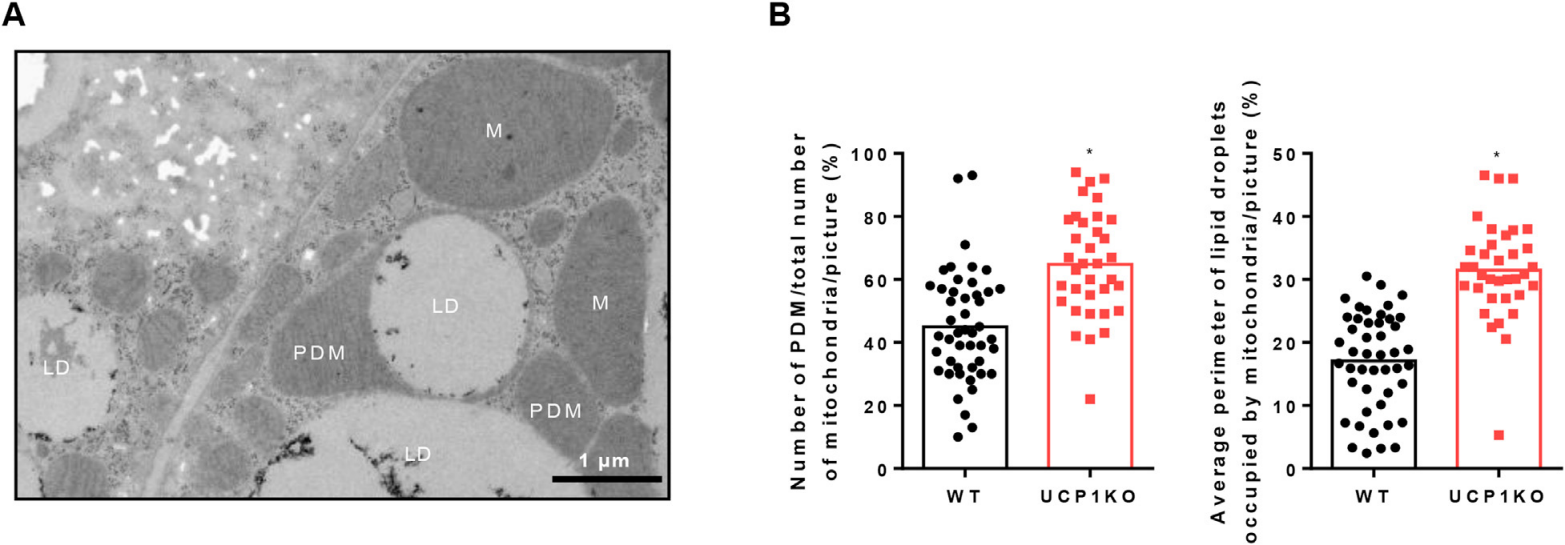
Brown UCP1-knockout adipocytes have a higher number of peridroplet mitochondria and mitochondria are tightly associated with lipid droplets. A) Representative electron micrograph of a fully differentiated 129Sv/S1 brown UCP1-knockout (UCP1KO) cell including lipid droplets, mitochondria, and peridroplet mitochondria (PDM). Scale bar represents 1 μm “LD” indicates a lipid droplet, “M” a mitochondrion, and “PDM” a peridroplet mitochondrion. B) (Left) Relative proportion of PDM in relation to the total number of mitochondria in 129Sv/S1 brown wild type (WT) and UCP1KO adipocytes. (Right) Relative proportion of lipid droplet perimeter occupied by mitochondria. n = 35–46 pictures from two independent biological experiments. A two-tailed t-test was applied. Asterisk (∗) indicates a significant difference between the two groups.

Although WT and UCP1KO adipocytes respond similarly to adrenergic stimulation by increasing their oxygen consumption and ramping up glucose and FA uptake, the respective underlying mechanisms causing these events are vastly different. Based on a recent publication, which revealed distinct mitochondrial populations in BAT specialized on carrying out defined functions [67], we explored the association between mitochondria and lipid droplets (Figure 5A). Mitochondria bound to lipid droplets, peridroplet mitochondria (PDM), show considerable resemblance to cytoplasmic mitochondria (CM) but differ by having a higher ATP synthesis capacity to support TG synthesis and lipid droplet expansion [67]. Even in the basal state, almost two third of all mitochondria in brown UCP1KO adipocytes are associated with lipid droplets compared to only slightly more than 40% in WT cells (Figure 5B). Additionally, not only the frequency but also the quality of the association was stronger in UCP1KO cells, since on average around 30% of total lipid droplet perimeter were occupied by mitochondria as opposed to less than 20% in WT adipocytes.

### 3.5 UCP1KO mice recruit a futile cycle of lipolysis and re-esterification for thermogenesis

We finally addressed whether the futile lipid cycle occurred *in vivo* and if so, would it be recruited in response to cold exposure. Therefore, WT and UCP1KO mice were stepwise acclimated to different ambient temperatures: 30 °C (warm-acclimated, WA), 20 °C (mild cold-acclimated, MCA), or 6 °C (cold-acclimated, CA) over at least three weeks. After the acclimation period, neither genotype nor housing temperature affected body weight of the animals, but the weight of individual adipose tissue depots largely differed (Table 3). Compared to the WA condition, weight of eWAT and iWAT depots in WT mice progressively decreased in MCA and CA. In UCP1KO mice, eWAT responded similarly, while iWAT mass was not significantly reduced. In fact, in UCP1KO mice at CA, iWAT was significantly heavier than in their WT counterparts. Mass of iBAT of WT mice was lower at MCA, while it was comparable at WA and CA. On the contrary, iBAT of UCP1KO mice expanded in CA, with a 2-fold and 1.3-fold elevation of mass in MCA and CA, respectively (Table 3). This difference in mass was partly due to a higher protein and TG content of UCP1KO iBAT (Supp. Figure 2D) and might also be linked to altered glycogen storage. These results indicate different and depot-specific adaptations to cold exposure in WT and UCP1-ablated mice. In summary, MCA and CA UCP1KO mice specifically recruit iBAT mass and preserve iWAT mass.

**Figure 6:**
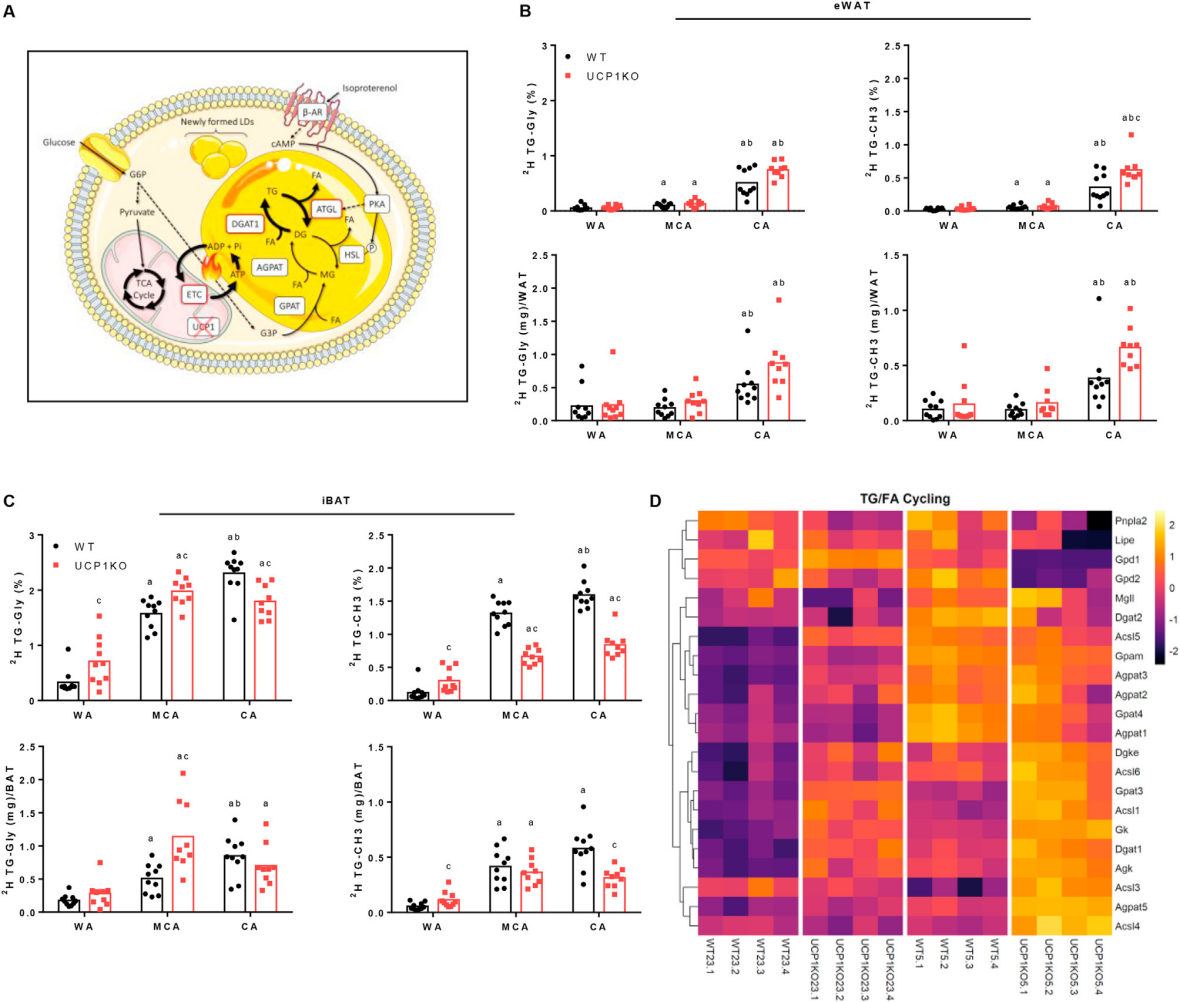
Futile lipid cycling is recruited for non-shivering thermogenesis in adipose tissues of UCP1-knockout mice. A) Graphical summary of futile triglyceride/fatty acid (TG/FA) cycling in adipocytes. In response to a cold sensation, norepinephrine, here mimicked by isoproterenol, binds to adrenergic receptors and triggers the downstream cAMP-PKA-signalling cascade, which leads to the activation of lipolysis. ATGL and HSL-mediated hydrolysis of TGs and diglycerides (DGs) causes an immediate increase in cellular FA levels. The majority of newly released FA in brown UCP1KO adipocytes is re-esterified onto DGs and possibly to a lesser extent onto monoglycerides (MGs) as well as G3P. In this setting, more glucose is taken up to replenish the TCA cycle, maintain protonmotive force, and most importantly to provide G3P backbones serving as FA acceptor molecules. Primarily DGAT1 but potentially also other acyltransferases, such as glycerol-3-phosphate acyltransferases (GPATs) and 1-acylglycerol-3-phosphate O-acyltransferase (AGPATs), catalyze the esterification of FA derived from intracellular TG stores or taken up from the circulation, which is preceded by the ATP-dependent activation. The breakdown of TGs is simultaneously counterbalanced by re-synthesis, and thus lipid flux in both directions accelerates causing ATP turnover to increase without altering metabolite levels. Thereby, ATP consumption, its anaerobic glycolytic provision, and generation of ATP *via* oxidative phosphorylation directly linked to the flux through the mitochondrial electron transport chain (ETC) can be adjusted by enhancing or reducing lipid flux, which represents the theoretical foundation of TG/FA cycling. This figure was created using Servier Medical Art templates, which are licensed under a Creative Commons Attribution 3.0 Unported License; https://smart.servier.com. B) Futile TG/FA cycling activity and *de novo* lipogenesis in epididymal white adipose tissue (eWAT) of C57BL/6J wild type (WT) and UCP1-knockout (UCP1KO) mice acclimated to different ambient temperatures: 30 °C “warm-acclimated” (WA), 20 °C “mild cold-acclimated” (MCA), or 6 °C “cold-acclimated” (CA), for at least three weeks. n = 9–10 animals from two independent experiments. Relative enrichment is shown in top panels; enrichment per depot considering total tissue weight (Table 3) is shown in bottom panels. A two-way ANOVA followed by Tukey's HSD was applied. “a” indicates a significant difference from the WA group of the respective genotype. “b” indicates a significant difference from the MCA group of the respective genotype. “c” indicates a significant difference between the two genotypes within one treatment level. C) Futile TG/FA cycling activity and *de novo* lipogenesis in interscapular brown adipose tissue (iBAT) of WA, MCA, and CA C57BL/6J WT and UCP1KO mice. n = 9–10 animals from two independent experiments. Relative enrichment is shown in top panels; enrichment per depot considering total tissue weight (Table 3) is shown in bottom panels. A two-way ANOVA followed by Tukey's HSD was applied. “a” indicates a significant difference from the WA group of the respective genotype. “b” indicates a significant difference from the MCA group of the respective genotype. “c” indicates a significant difference between the two genotypes within one treatment level. D) Regulation of proteins potentially involved in TG/FA cycling in iBAT of C57BL/6N WT and UCP1KO mice acclimated to 23 °C or 5 °C. The complete set of processed mass spectrometry data can be found in Supp. Table 4. n = 4 animals. Column labels represent individual mice: WT (WT23) and UCP1KO (UCP1KO23) mice acclimated to 23 °C, WT (WT5) and UCP1KO (UCP1KO5) mice acclimated to 5 °C. Row labels represent gene symbols of proteins. Normalized protein intensities were scaled by calculating z-scores for each protein. Cell color indicates z-score. Abbreviations: Long-chain acyl-CoA synthetase (Acsl), acylglycerol kinase (Agk), 1-acylglycerol-3-phosphate O-acyltransferase (Agpat), diacylglycerol O-acyltransferase (Dgat), diacylglycerol kinase epsilon (Dgke), glycerol kinase (Gk), mitochondrial glycerol-3-phosphate acyltransferase (Gpam), glycerol-3-phosphate acyltransferase (Gpat), glycerol-3-phosphate dehydrogenase (Gpd), hormone-sensitive lipase (Lipe), monoglyceride lipase (Mgll), adipose tissue triglyceride lipase (Pnpla2).

To assess TG/FA cycling activity in adipose tissue *in situ*, mice were treated with ^2^H_2_O, and the incorporation of ^2^H into glycerol and FA methyl moieties of TGs was measured [17,18]. ^2^H enrichment of glycerol is determined by the rates of glycolytic provision of G3P and glyceroneogenesis. Incorporation of ^2^H-glycerol in the TG fraction of cellular lipids (^2^H TG-Gly %) depends directly on FA esterification, and therefore could serve as a qualitative surrogate marker for TG/FA cycling rates. ^2^H enrichment in FA methyl (CH_3_) chains specifically represents FA DNL, because ^2^H can only be incorporated during the reaction catalyzed by fatty acid synthase but not during FA elongation or desaturation. In general, we did not expect major differences between genotypes at thermoneutrality, because thermogenic requirements should be minimal or zero. However, we hypothesized that with decreasing temperature UCP1KO mice would substantially ramp up TG/FA cycling rates and primarily in iBAT (Figure 6A). Furthermore, we hypothesized that irrespective of the genotype, DNL in eWAT could support thermogenesis in iBAT, which may become even more important at lower temperatures [17]. Since we noticed sometimes very large differences in depot size between genotypes at different temperatures (Table 3), we additionally calculated an overall enrichment rate per depot, ^2^H TG-Gly and ^2^H TG-CH3 (mg)/adipose tissue, by including tissue weights.

In eWAT ^2^H enrichment of glycerol and FAs in TGs was similarly low in WA mice of both genotypes (Figure 6B). TG/FA cycling activity as well as DNL gradually increased with cold adaptation, while overall the largest increment was observed from MCA to CA. When mice were housed at 6 °C, DNL activity was significantly higher in UCP1KO mice and we could detect a trend for more TG/FA cycling in the absence of UCP1. Collectively, these data suggest that increased lipid cycling in eWAT, partly supported by DNL, may contribute to adaptive thermogenesis. The apparent compensatory upregulation in the absence of UCP1-mediated thermogenesis could reflect enhanced *in situ* thermogenesis resulting from futile lipid cycling, and elevated efflux of FAs into the circulation serving a dual purpose as energy fuel for thermogenesis or as a substrate for TG/FA cycling in other tissues. Given the strong reduction in eWAT mass upon cold exposure and the tissue's relatively low oxidative capacity, the latter option might be of higher importance for whole-body energy metabolism and thermogenesis [17]. Gene expression profiling supports this notion. Thus, a strong upregulation of genes related to FA biogenesis, such as fatty acid synthase (*Fas*) and pyruvate carboxylase (*Pc*), TG synthesis (*Dgat2*), and the release of FAs from lipid droplets (*Atgl* and *Cidea*) in eWAT of UCP1KO mice was observed (Supp. Table 1). Changes in the expression of genes putatively linked to re-esterification (*Dgat1* and *Gpd1*) of FAs were relatively small.

Nevertheless, in iBAT relative incorporation of ^2^H into glycerol and FAs of TGs was severalfold higher compared to eWAT (Figure 6C). TG/FA cycling and DNL in iBAT still increased in a dose-dependent manner upon cold exposure in mice of both genotypes. In contrast to eWAT, the difference between WA and MCA animals was by far the most pronounced, whereas the difference between MCA and CA animals regarding ^2^H enrichment was smaller. This indicated that lipid cycling activity was higher in WA and MCA UCP1KO mice in comparison to the corresponding WT mice. However, enrichment did not differ between MCA and CA UCP1KO mice translating into a lower ^2^H incorporation into glycerol of TGs in iBAT of CA UCP1KO as compared to CA WT mice. If enrichment is expressed per whole depot, the difference becomes even greater between MCA WT and UCP1KO animals and negligible in CA animals (Figure 6C). Interestingly, WT mice massively increased DNL during cold exposure, while we could only detect a very modest upregulation in UCP1KO mice. Therefore, we concluded that UCP1KO mice preferentially recruit a futile cycle of lipolysis and re-esterification of FAs in brown adipocytes for adaptive thermogenesis as compared with WT mice. Moreover, in the absence of UCP1, NST in iBAT may rather depend on exogenous substrates and possibly lipoprotein lipase (LPL)-mediated uptake of FAs from circulating TGs, because TG stores were largely preserved despite a very modest increase in DNL.

**Figure 7:**
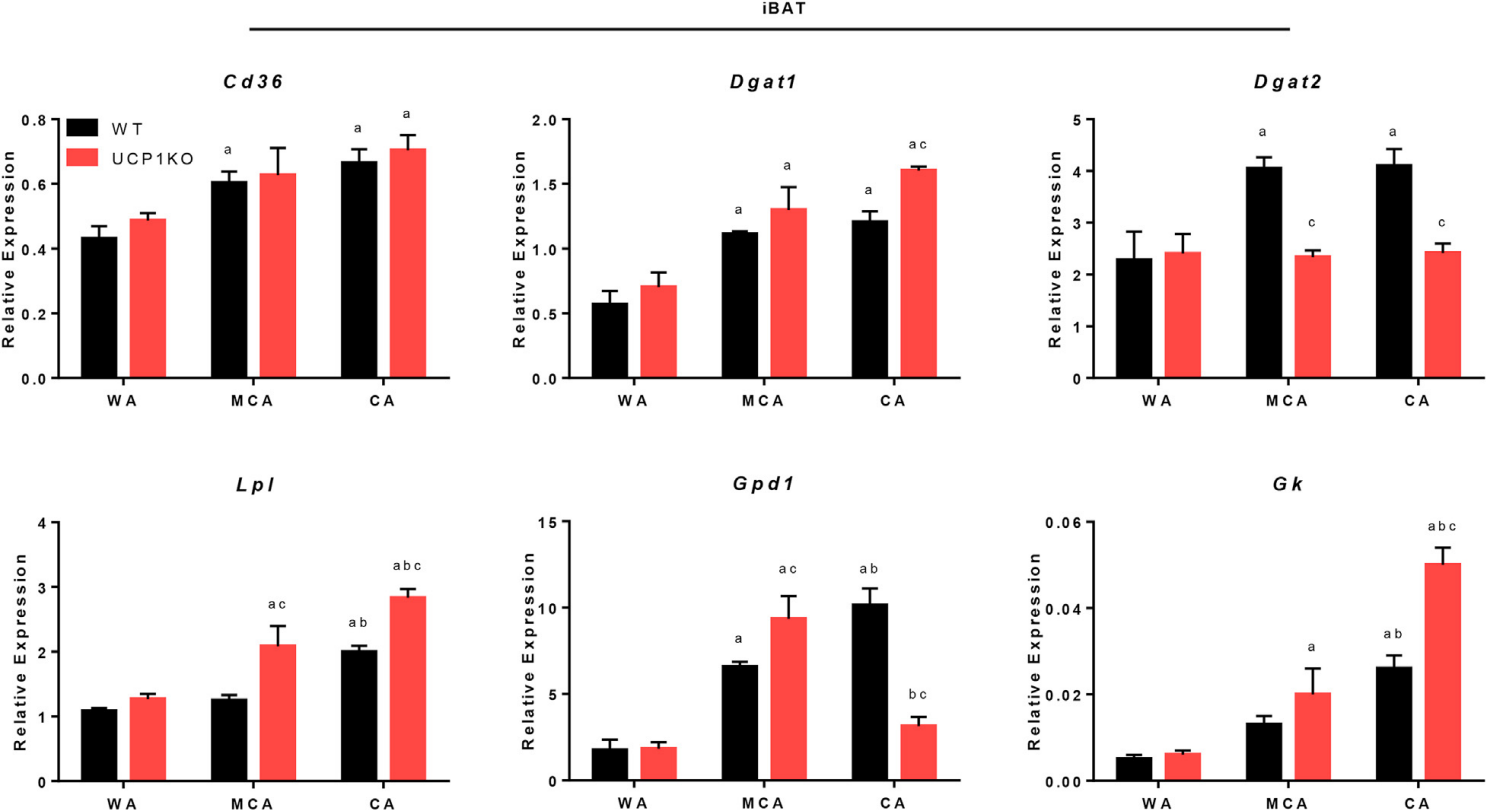
Expression of selected genes in iBAT of C57BL/6J wild type (WT) and UCP1-knockout (UCP1KO) mice acclimated to different ambient temperatures: 30 °C “warm-acclimated” (WA), 20 °C “mild cold-acclimated” (MCA), or 6 °C “cold-acclimated” (CA), for at least three weeks. n = 4–5 animals (data from one experiment; confirmed in two independent experiments). “a” indicates a significant difference from the WA group of the respective genotype. “b” indicates a significant difference from the MCA group of the respective genotype. “c” indicates a significant difference between the two genotypes within one treatment level. Abbreviations of genes: CD36 molecule (*Cd36*), diacylglycerol O-acyltransferase 1 (*Dgat1*), diacylglycerol O-acyltransferase 2 (*Dgat2*), glycerol kinase (*Gk*), glycerol-3-phosphate dehydrogenase 1 (*Gpd1*), lipoprotein lipase (*Lpl*).

In line with our hypothesis that energy stores in eWAT are sacrificed to supply other thermogenic tissues, such as iBAT and iWAT, non-esterified FA (NEFA) concentrations in the plasma of CA UCP1KO mice were drastically reduced in response to cold exposure (Table 3) while at the same time Cd36 expression in iBAT, a protein facilitating the uptake of FAs into cells, was upregulated (Figure 7). Moreover, *Lpl* transcript levels in iBAT of CA UCP1KO mice were positively regulated by cold exposure and significantly higher than in CA WT mice. Concurrently, plasma TG levels in UCP1-ablated mice were significantly reduced by 50% compared to their WT counterparts clearly suggesting that in the absence of UCP1 there is a higher demand for FAs derived from classical white adipose tissue [17,68,69] and TG-rich lipoproteins originating from hepatocytes or enterocytes could support UCP1-independent thermogenesis in iBAT and potentially also iWAT (Table 3). As expected, *Dgat1* expression was significantly increased in iBAT of MCA and CA mice, with the largest incremental increase in CA UCP1KO mice (Figure 7). This underlines the importance of DGAT1-mediated re-esterification directly linked to TG lipolysis [60], whereas *Dgat2* levels were unaffected from cold exposure in UCP1-ablated mice.

*Gpd1* levels were markedly increased in MCA UCP1KO animals, while a further reduction in ambient temperature caused Gpd1 expression to drop again (Figure 7). Interestingly, gene expression of glycerol kinase (*Gk*) stepwise increased with a decreasing ambient temperature. This was more pronounced in mice lacking UCP1 and MCA was already sufficient to induce a significant upregulation, as reported previously [27,70]. Other than serum lipids, plasma glucose levels were neither affected by the housing temperature nor by the genotype, but insulin concentrations were significantly elevated in CA UCP1KO mice (Table 3). Increased insulin secretion may funnel more substrates, such as amino acids, FAs, and glucose, into insulin-responsive tissues and it could counteract the extensive catabolism of thermogenic adipose tissues elicited by continuously high sympathetic stimulation. Therefore, the amount of nutrients supplied to iBAT and iWAT *via* the circulation may disproportionately grow as soon as intracellular energy stores are significantly depleted. In line with this assumption, we found higher gene expression levels of glycogen phosphorylase (*Pyg*l), the enzyme mediating glycogen breakdown, in iBAT of cold-exposed WT animals, while only MCA but not CA UCP1KO mice responded in a similar way potentially indicating that glycogen stores were already exhausted when housed at 6 °C (Supp. Table 1). Since transcript levels do not necessarily correlate well with protein levels, we also examined cold-induced adaptations with respect to proteins involved in TG/FA cycling (Figure 6D), other futile substrate cycles (Supp. Figure 3C), and lipid metabolism (Supp. Figure 3D) in iBAT of WT and UCP1KO mice in a separate cohort using an additional UCP1-ablated mouse model. At the protein level, detected changes specifically with respect to enzymes responsible for activation and esterification of FAs (Figure 6D) as a function of temperature and genotype were in good agreement with our initial hypothesis and previous observations and further corroborated the theory that mice lacking UCP1 recruit futile lipid cycling for NST. Although our functional *in vitro* data at least argue against an involvement of futile Ca^2+^ cycling, we also found an induction of proteins associated with Ca^2+^ and creatine cycles (Supp. Figure 3C), but we cannot assess whether they significantly contribute to heat production *in vivo* and whether flux rates are in fact increased.

Furthermore, it is extremely interesting that the depletion of the respiratory chain in iBAT of MCA and CA UCP1KO mice was not as severe as previously described [71]. We detected small morphological changes and a few mitochondria with aberrant cristae morphology in UCP1KO iBAT (Supp. Figure 2E). However, these defects appear to be minor at room temperature and only a small subset of mitochondria is affected. At the same time, only NDUFA9 (complex I) and COX4 (complex IV) were reduced (Supp. Figure 3A). Additionally, ATP5A1 protein levels, a subunit of the catalytic domain of the mitochondrial ATP synthase (complex V), were even 1.5-fold and 2-fold higher in MCA and CA UCP1-ablated mice, respectively. Since the futile TG/FA cycle only depends on ATP synthesis and turnover, a decreased amount of complex I to IV would not preclude UCP1-independent thermogenesis as long as their capacity is high enough to cover the dissipation of protonmotive force by a fully active ATP synthase. Therefore, we have good reason to believe that brown fat of cold-acclimated UCP1KO mice is equipped with the electron transport chain machinery required to substantially contribute to NST.

Taken together, supported by several lines of evidence, we demonstrate that UCP1KO mice, and to a lesser extent WT mice, enhance TG/FA cycling to defend their body temperature in the cold. In the absence of UCP1, additional futile lipid cycling capacity is not only provided by expanding iBAT mass, but we also detected a very characteristic cold-induced transcript and proteome signature in iBAT and eWAT. These integrated changes in adipose tissue metabolism most likely ensure sufficient substrates for oxidation in iBAT, readily available and matching amounts of glycerol backbones and FAs, and the enzymatic machinery required to orchestrate all reactions involved.

## 4 Discussion

In the present study, we investigated potential mechanisms of UCP1-independent heat generation in murine BAT. We identified a futile substrate cycle of lipolysis and FA re-esterification as the major source of NST in brown adipocytes of UCP1KO mice. The underlying theoretical framework that futile substrate cycles and in particular futile lipid cycling could contribute to NST was proposed 60 years ago [54]. Since then, the topic has been picked up from time to time but meanwhile sank into obscurity again, aside from a few exceptions. Only recently futile cycles experience a renaissance. For decades it has been known that adipocytes with low UCP1 levels, i.e. white adipocytes, and since more recently also brown and white UCP1KO adipocytes, increase their oxygen consumption in response to adrenergic stimulation [30,54,55,72,73]. Scientists hypothesized that FAs released during lipolysis may either undergo ATP dependent re-esterification or degradation through beta-oxidation, or may cause beneficial mild mitochondrial uncoupling. However, the exact mechanism of this phenomenon has never been fully resolved, as the molecular evidence provided was rather anecdotal.

Here we demonstrated that beta-adrenergically stimulated respiration rates of brown UCP1-knockout adipocytes are fully sensitive to inhibition of the mitochondrial ATP synthase. Potential mild non-specific uncoupling due to excess FAs [74,75] or cyclosporin-sensitive opening of the mitochondrial permeability transition pore [76,77], as reported previously [55,72], could be excluded. Our results prove that mitochondrial beta-oxidation is not in any way part of the futile TG substrate cycle, other than as a source of reducing equivalents. After all, we pinpointed a set of key enzymes in TG/FA cycling on a cellular level and elucidated the role of glucose. A few open questions demand a critical appraisal of our key findings.

Pharmacological inhibition of long-chain acyl-CoA synthetase activity (triacsin C), i.e. ATP-dependent activation of FAs, did not fully abolish oxygen consumption linked to futile TG cycling. This suggests that either the enzyme was not completely inhibited, since a high cellular lipid content [78,79] and bovine serum albumin-buffered assay medium are strongly affecting substance partitioning and availability [80], or different isoforms [81] or even short and medium-chain acyl-CoA synthetases also contribute to the activation of FAs prior to re-esterification during active lipolysis. Interestingly, triacsin C was ultimately even slightly more potent than oligomycin in decreasing free FA (FFA) re-esterification rates. Although oligomycin can fully reverse the isoproterenol-induced increase in oxygen consumption, a certain amount of FAs may still be activated and re-esterified as long as glycolytic ATP production can compensate for the inhibition of the mitochondrial ATP synthase. Similarly, combining both DGAT inhibitors did not fully prevent increased respiration rates following adrenergic stimulation. This may be explained by the activity of different acyltransferases, such as glycerol-3-phosphate acyltransferases (GPATs) and 1-acylglycerol-3-phosphate O-acyltransferase (AGPATs), which may also catalyze re-esterification of FAs during active lipolysis. However, specific and well characterized inhibitors targeting these enzymes are not available at the moment. Thus, it still has to be resolved if this futile substrate cycle involves the whole TG synthesis pathway from G3P to TGs or if it is predominantly restricted to di- and triglycerides. Since futile lipid cycling activity apparently depends on glycolytic provision of G3P *in vitro*, the first steps of lipid synthesis might also have a greater importance than initially assumed.

Apart from this, the absence of UCP1 may have even more far-reaching consequences, since we found clear differences between WT and UCP1KO cells with respect to the interaction between mitochondria and lipid droplets. Therefore, it is enticing to speculate that this shift in mitochondrial subpopulations at least partly supports the observed phenotypic differences. Brown WT adipocytes rely on UCP1 mediated thermogenesis, and thus would require less PDM and more CM, as CM are specialized on beta-oxidation fueling thermogenesis. On the contrary, UCP1KO adipocytes have an increased ATP demand due to higher rates of FA re-esterification favoring the occurrence of PDM, which may facilitate heat generation by futile lipid cycling. Monitoring the dynamic interaction between mitochondria and lipid droplets upon adrenergic stimulation, or during states of impaired lipid cycling will clearly show, if the emergence of certain mitochondrial populations correlates with futile cycling activity, lipid turnover and ATP consumption.

Finally, we corroborated these new mechanistic insights with quantitative data on TG/FA cycling in classical white and brown adipose tissue of WT and UCP1KO mice. The Kozak UCP1KO mouse [26] has made a great contribution to uncovering the mechanism behind NST in BAT and characterizing its physiological significance [[28], [29], [30], [31],[82], [83], [84]]. Based on the substantially reduced effect of norepinephrine on whole body oxygen consumption in UCP1-ablated mice [82] or in adipocytes isolated from BAT of these animals [84], it was concluded that only UCP1 mediates adaptive adrenergically-mediated NST [85]. However, with the application of magnetic resonance (MR) imaging to quantify dynamic changes in lipid content of BAT as a surrogate measure of NST, Grimpo and coworkers demonstrated that UCP1KO mice show a thermogenic response to norepinephrine in iBAT. In stark contrast to the previous publications mentioned before, the MR imaging approach indicated that this response was even stronger in cold-acclimated animals, clearly suggesting an adaptive recruitment of NST capacity in mice lacking UCP1 [32]. In a recent publication using MR thermometry, scientists observed a significant increase in iBAT temperature of UCP1KO mice following adrenergic stimulation, which was evidently preceding the rise in rectal body temperature [33]. Moreover, it has been shown that acute beta3-adrenergic stimulation induced glucose uptake in BAT independently of UCP1 and whole-body oxygen consumption [64]. These observations are thus compatible with our results presented in this study suggesting adrenergic stimulation of UCP1-independent NST in BAT. Interestingly, a study in rats treated with rosiglitazone gave very similar results. An upregulation of PPARγ-responsive genes, including Gpats and specifically Dgat1, lead to 2.2-fold increase in TG/FA cycling activity at room tenmperature [86], whereas in fact, TG/FA cycling in iBAT of UCP1KO mice has never been measured before.

Our results support the idea that TG/FA cycling and DNL in white adipose tissue, together with hepatic very-low-density lipoprotein TG synthesis, may be involved in controlling blood lipid levels and providing FAs as fuel for thermogenesis in iBAT and possibly other tissues during cold exposure [17,32]. Thus, the induction of both TG/FA and DNL activities in eWAT of CA UCP1KO mice is in agreement with the induction of cytochrome c oxidase observed in eWAT of UCP1KO but not WT mice acclimated to cold [28]. Here, we also observed such a genotype-specific pattern regarding the induction of TG/FA cycling and DNL in iBAT. Interpretation of these data in iBAT must take changes of depot mass into consideration, because it is differentially affected by cold acclimation depending on the genotype (Table 3). The significant increase in futile lipid cycling activity observed in MCA UCP1KO mice was even greater when expressed per whole depot. In the attempt to estimate whole depot capacity, it is probably not sufficient to merely look at depot size to properly account for effective genotype-dependent metabolic changes in iBAT, especially since protein and TG content are differently affected. In fact, we most likely underestimated TG/FA cycling activity in iBAT of MCA and CA UCP1KO mice with the true capacity being considerably larger. Directly related, there is a conceptual limitation of our study pertaining to the determination of futile lipid cycling activity. The D_2_O-based labelling approach is only able to capture a certain defined fraction of the whole futile cycle, as FAs, which are released from tri – and diglycerides during active lipolysis and re-esterified onto di – or monoglycerides originating from the lipolytic process itself, will not be included in the analysis. At least based on our *in vitro* data, this particular ATGL-DGAT1-mediated subcycle accounts for the largest share of newly released and subsequently re-esterified FAs in brown UCP1KO cells, which could also contribute to the fact that *in vivo* the measured difference between WT and UCP1KO mice was smaller than we would have suspected.

Importantly, the analysis based on *in vivo* D_2_O-labelling of tissue lipids includes only G3P backbones provided *via* glycolysis and glyceroneogenesis, while G3P derived from GK activity is not, which makes interpretation of enrichment data more challenging. This does not pose a problem for white adipose tissue, i.e. eWAT, where GK activity is negligible. For iBAT, however, there is a growing body of evidence suggesting considerable GK activity in iBAT and beige adipose tissues [27,70,87,88]. Cold exposure induces GK expression specifically in thermogenic adipose tissues. In line with previous studies [27,70], we found a more pronounced cold-stimulated induction in iBAT of UCP1-ablated mice. Consequently, TG/FA cycling rates in UCP1KO mice during cold exposure may be in fact much higher than initially suspected and the effect of UCP1 ablation could be even more substantial. Additionally, even if total G3P production remains unchanged, switching the source of G3P from glycolysis to GK, regardless of the underlying cause, would be misinterpreted as a decrease in TG/FA cycling activity. In line with that, we found that GPD1 transcript and protein levels are lower, and GK levels are significantly higher in iBAT of UCP1KO mice at lower temperatures and expression of these two proteins follows a completely different pattern compared to their WT counterparts. This could indicate that mild cold stress still allows UCP1KO mice to use G3P as substrate for oxidation and backbone for re-esterification. However, severe cold stress and the concomitant elevated energetic demand may *de facto* exclude the metabolic inefficiency connected to the conversion of glucose to G3P. Consequently, CA UCP1KO mice may provide as little as possible but as much as required G3P *via* glycolysis, while the importance of regenerating G3P through GK activity drastically grows [88]. We speculate that in this scenario, the majority of glucose is metabolized to pyruvate and fully oxidized supporting ATP synthesis *via* oxidative phosphorylation, and only a minor fraction is converted to G3P.

At this point, it should be mentioned that a few studies indeed attribute a lower metabolic flexibility and a numerically slightly higher respiratory quotient to CA UCP1KO mice [28,29]. These findings may indicate that cold acclimated mice lacking UCP1 have temporarily higher glucose oxidation rates and that a switch in the fuel source needs to be controlled even more tightly and carefully balanced. Thus, we assume that in iBAT of CA UCP1KO mice GDP1-mediated G3P production only has to cover FAs, which stoichiometrically exceed the amount of available glycerol backbones provided by lipolysis and GK as well as LPL activity. This in turn, along with the different depot sizes, could also explain why CA UCP1KO mice apparently did not increase the ^2^H-enrichment-based lipid cycling rate further. Overall, we have good reason to believe that in fact true TG/FA cycling activity in MCA and CA UCP1KO mice may have very likely been much higher. Addressing the issue of GK activity in different mouse models across varying ambient temperatures would require the employment of labelled glycerol and the subsequent detection of the incorporation rate into TGs. While that would still be within the realm of possibility, specific quantification of the ATGL-DGAT1-subcycle is close to impossible, as this would require the use of multiple stable isotope labels in parallel and extremely sophisticated spectra analysis. Furthermore, the unknown contribution of GK is a limitation possibly also affecting the measured *in vitro* re-esterification rates. Since we did not determine GK activity in cultured adipocytes, we cannot rule out a genotype-dependent difference. Thus, calculated cycling rates based on the release of glycerol and FAs might be skewed. Nevertheless, the published literature and our own data consistently demonstrated higher GK expression in UCP1KO BAT and brown adipocytes lacking UCP1 suggesting a higher GK activity, but this remains to be tested.

Moreover, we would like to point out that the mechanistic cell culture data might not necessarily be transferred directly to the *in vivo* situation. Cell culture medium contains high amounts of glucose and cultured adipocytes have considerable intracellular glycogen and lipid stores, while there is no extracellular source of FAs, TGs, and glycerol as in the *in vivo* situation. In contrast, in mice, a considerable amount of substrates and metabolites is transported to iBAT *via* the blood, whereas intracellular nutrient stores, such as lipid droplets and in particular glycogen, may be substantially depleted during prolonged cold exposure. In addition, we are only looking at a short period (minutes or hours) after acute activation of TG/FA cycling in the cell model. In the animal model, however, a longer acclimation period (weeks) is obligatory, and therefore in this case we are rather studying a chronic process that may have been preceded by other acute changes. These small differences could nevertheless be crucial and explain why glucose might have a slightly different intracellular fate in cultured adipocytes and why ATGL-mediated lipolysis and glycerol recycling by GK might have a different importance in cultured brown adipocytes than in iBAT of mice. Thus, the brown adipocyte cell model helped us to understand the operation of TG/FA cycling in the context of the intracellular metabolic pathways involved in thermogenesis. The animal experiments enabled us to demonstrate the role of TG/FA cycling in BAT in the integrated whole body response to cold acclimation, i.e. the gradual increase of futile lipid cycling activity with lowering the acclimation temperature. This clearly indicates the adaptive nature of TG/FA cycling-based UCP1-independent thermogenesis in BAT. Its quantitative contribution to whole-body thermogenesis may be substantial, since it reflects the oxidative capacity of BAT. Moreover, our results are in agreement with the notion that the TG/FA cycling-based thermogenesis in BAT may depend on the inter-organ cycling of FAs including adipose tissues and liver [17].

Lastly, we want to stress that only male mice were used for the *in vitro* studies obviously limiting the translational potential, since we already know that overall lipoprotein and lipid metabolism is strongly affected by sex (hormones) and the menstrual cycle [89]. This interesting aspect deserves more future attention, especially because for the widely studied inbred mouse strains C57BL/6J and C57BL/6N, males are more susceptible to diet-induced obesity than females, and just recently sex-specific differences in the browning propensity of certain adipose tissue depots were reported [90,91]. Consequently, it would not be entirely implausible that UCP1-independent energy consuming mechanisms, such as futile substrate cycles including TG/FA cycling, are enhanced specifically in female mice, but this remains to be investigated.

Despite these limitations, we could clearly demonstrate that futile lipid cycling in iBAT is part of adaptive thermogenesis and that this process possibly in combination with other thermogenic mechanisms is specifically recruited in the absence of UCP1. Intracellular energy stores, energy substrates from other adipose tissues and organs, as well as food-born lipids and carbohydrates are delivered *via* the circulation to iBAT and fuel UCP1-independent thermogenesis. Previous work has already indicated an important role for adipose tissue TG/FA cycling in enhancing energy expenditure by stimulating ATP turnover [11,[16], [17], [18],^23^,^92^,^93^]. We are currently focusing on the question whether this process is even more complex *in vivo* and whether it has more unforeseen implications beyond the proposed thermogenic properties especially in terms of the dynamic interaction between mitochondria and lipid droplets. Additionally, further studies addressing the inter-organ part of lipid cycling and its importance for thermogenesis are needed [19]. In the future, generation of new mouse models with conditional tissue-specific deletions of enzymes involved in FA re-esterification and TG synthesis will enable novel insights into the contribution of TG/FA cycling to NST, total energy expenditure, and susceptibility to diet-induced obesity.

## Author contributions

J.O. conceptualization, performed experiments, data analysis, interpretation of data, visualization, supervision, writing - original draft; P.J. acquired funding, conceptualization, performed experiments, data analysis, interpretation of data, visualization, supervision; K.B., O.K., T.G., T.F., S.Schw., and Y.L. conceptualization, interpretation of data; S.D. conceptualization, performed animal experiment, interpretation of data; P.G. generated proteome data, data analysis, interpretation of data; B.K. data analysis, interpretation of data; S.B., M.S.H. performed experiments; J.E. acquired funding, conceptualization, interpretation of data; S.Schm. generated electron micrographs, data analysis, interpretation of data; H.Z. conceptualization, interpretation of data; K.A. conceptualization, performed animal experiment, gene expression analysis, data analysis; R.P. NMR analysis; J.F. extraction of TG for NMR analysis; P.Z. introduction of the NMR method; J.K. conceptualization, interpretation of data, supervision, writing - review & editing; M.K. acquired funding, conceptualization, interpretation of data, supervision, writing - review & editing.
