# Supplementary material for: Loss of UCP1 function augments recruitment of futile lipid cycling for thermogenesis in murine brown fat
Source: Mol Metab. 2022 Apr 22;61:101499. doi: 10.1016/j.molmet.2022.101499 (PMC9097615; doi:10.1016/j.molmet.2022.101499)
Supplement: Multimedia component 4 [file mmc4.docx]

# Supplement

Supplementary Figure 1: The effect of acute adrenergic stimulation on the cellular proteome of brown wild type (WT) and UCP1-knockout (UCP1KO) adipocytes.

A) Two-dimensional annotation enrichment analysis showing cellular components, which are significantly regulated upon adrenergic stimulation in at least one of the two genotypes. Components that are preferentially upregulated in stimulated UCP1KO adipocytes and downregulated or not affected in WT cells are located above the x-axis and near or to the left of the y-axis. Values between 0 < x ≤ 1 indicate an upregulation after the addition of isoproterenol (Iso), whereas values between -1 ≤ x < 0 indicate a downregulation. Terms related to futile substrate cycles are highlighted in red. Not all terms are displayed due to overlapping points. The complete set of pathways can be found in Supp. Table 3. n = 5 independent biological experiments.

B) – D) Change of expression levels of selected proteins in primary cultures of 129Sv/S1 WT and UCP1KO adipocytes in response to an adrenergic stimulus. The complete set of processed mass spectrometry data can be found in Supp. Table 2. n = 5 independent biological experiments. Asterisk (*) indicates a significantly regulated protein in response to the treatment within one genotype. Proteins are designated by their gene name. Abbreviations: Patatin like phospholipase domain containing 2 (Pnpla2), lipase E hormone sensitive type (Lipe), monoglyceride lipase (Mgll), acyl-CoA synthetase long chain family member 1 (Acsl1), acyl-CoA synthetase long chain family member 3 (Acsl3), acyl-CoA synthetase long chain family member 4 (Acsl4), ELOVL fatty acid elongase 1 (Elovl1), acyl-CoA desaturase 1 (Scd1), acyl-CoA desaturase 2 (Scd2), carnitine palmitoyltransferase 1B (Cpt1b), glycerol kinase (Gk), diacylglycerol kinase alpha (Dgka), diacylglycerol kinase delta (Dgkd), diacylglycerol kinase epsilon (Dgke), diacylglycerol kinase theta (Dgkq), 1-acylglycerol-3-phosphate O-acyltransferase 1 (Agpat1), 1-acylglycerol-3-phosphate O-acyltransferase 2 (Agpat2), 1-acylglycerol-3-phosphate O-acyltransferase 3 (Agpat3), 1-acylglycerol-3-phosphate O-acyltransferase 4 (Agpat4), 1-acylglycerol-3-phosphate O-acyltransferase 5 (Agpat5), glycerol-3-phosphate acyltransferase 4 (Agpat6), glycerol-3-phosphate acyltransferase 3 (Agpat9), phospholipid phosphatase 3 (Ppap2b), diacylglycerol O-acyltransferase 1 (Dgat1), perilipin 1 (Plin1), perilipin 2 (Plin2), perilipin 3 (Plin3), perilipin 4 (Plin4), perilipin 5 (Plin5), acetyl-CoA carboxylase alpha (Acaca), acetyl-CoA carboxylase beta (Acacb), ATP citrate lyase (Acly), fatty acid synthase (Fasn), fatty acid binding protein 4 (Fabp4), fatty acid binding protein 5 (Fabp5), solute carrier family 27 member 1 (Slc27a1), solute carrier family 27 member 3 (Slc27a3), solute carrier family 27 member 4 (Slc27a4), CD36 molecule (Cd36), MLX interacting protein like (Mlxipl), sterol regulatory element binding transcription factor 1 (Srebf1), insulin induced gene 1 (Insig1), cell death inducing DFFA like effector a (Cidea), cell death inducing DFFA like effector c (Cidec), mitochondrial calcium uniporter (Mcu), inositol 1,4,5-trisphosphate receptor type 1 (Itpr1), inositol 1,4,5-trisphosphate receptor type 2 (Itpr2), inositol 1,4,5-trisphosphate receptor type 3 (Itpr3), ATPase sarcoplasmic/endoplasmic reticulum Ca^2+^ transporting 1 (Atp2a1), ATPase sarcoplasmic/endoplasmic reticulum Ca^2+^ transporting 2 (Atp2a2), ATPase sarcoplasmic/endoplasmic reticulum Ca^2+^ transporting 3 (Atp2a3), creatine kinase B (Ckb), creatine kinase, M-type (Ckm), creatine kinase, mitochondrial 2 (Ckmt2), lactate dehydrogenase A (Ldha), lactate dehydrogenase B (Ldhb), glycerol-3-phosphate dehydrogenase 1 (Gpd1), glycerol-3-phosphate dehydrogenase 2 (Gpd2), pyruvate dehydrogenase E1 subunit alpha 1 (Pdha1), pyruvate dehydrogenase E1 subunit beta (Pdhb), pyruvate dehydrogenase complex component X (Pdhx), mitochondrial pyruvate carrier 1 (Mpc1), mitochondrial pyruvate carrier 2 (Mpc2), pyruvate carboxylase, mitochondrial (Pcx), phosphoenolpyruvate carboxykinase 1 (Pck1), phosphoenolpyruvate carboxykinase 2, mitochondrial (Pck2), 6-phosphofructo-2-kinase/fructose-2,6-biphosphatase 1 (Pfkfb1), 6-phosphofructo-2-kinase/fructose-2,6-biphosphatase 3 (Pfkfb3), glucose-6-phosphatase catalytic subunit 3 (G6pc3), glycogen phosphorylase B (Pygb), glycogen phosphorylase L (Pygl), glycogen phosphorylase, muscle associated (Pygm), phosphoglucomutase 1 (Pgm1,), phosphoglucomutase 2 (Pgm2), UDP-glucose pyrophosphorylase 2 (Ugp2), glycogenin 1 (Gyg), glycogen synthase 1 (Gys1), glycogen synthase 2 (Gys2), 1,4-alpha-glucan branching enzyme 1 (Gbe1).

B) Proteins associated with futile calcium and creatine cycling.

C) Proteins involved in lipid metabolism.

D) Proteins related to glucose metabolism.

Supplementary Figure 2: Beta-oxidation and permeability transition pore formation does not contribute to futile lipid cycling and UCP1-independent thermogenesis in brown adipocytes lacking UCP1, and glycolysis is dispensable for UCP1-mediated thermogenesis.

A) XF96 extracellular flux measurements of 129Sv/S1 brown UCP1-knockout (UCP1KO) adipocytes. Cells were pre-treated with etomoxir (100 µM final) for 1 h or with cyclosporin A (4.2 µM final) for 72 h. n = 14 – 23 wells from three independent biological experiments.

B) & C) XF96 extracellular flux measurements of primary cultures of fully differentiated 129Sv/S1 brown wild type (WT) adipocytes.

B) Cells were assayed in glucose-free medium. Glucose (25 mM final) or buffer was delivered via the second injection (port“B”) and the isoproterenol-induced increase in OCR was calculated. n = 14 – 16 wells from two independent biological experiments. A two-tailed t-test was applied. Asterisk (*) indicates a significant difference between the two groups.

C) Cells were pre-treated with 2-deoxyglucose (2DG, 50 mM final) or a combination of 2DG and pyruvate (2DG + Pyruvate; 2DG 50 mM final, pyruvate 5 mM final). Isoproterenol-induced increase in OCR was calculated. n = 24 – 37 wells from two independent biological experiments. One-way ANOVA followed by Tukey’s HSD was applied. “a” indicates a significant difference from the control group, and “b” from the 2DG group.

**D) Total protein and TG content per iBAT depot of C57BL/6J wild type (WT) and UCP1-knockout (UCP1KO) mice acclimated to different ambient temperatures: 30 °C “warm-acclimated” (WA), 20 °C “mild cold-acclimated” (MCA), or 6 °C “cold-acclimated” (CA), for at least three weeks. n = 4 – 5 animals (data from one experiment). “a” indicates a significant difference from the WA group of the respective genotype. “b” indicates a significant difference from the MCA group of the respective genotype. “c” indicates a significant difference between the two genotypes within one treatment level.**

**E)** **Representative electron micrographs of iBAT from adult 129Sv/S1 wild type (WT) and UCP1-knockout (UCP1KO) mice. Animals were group-housed at 23 °C and had *ad libitum* access to food and water °C. n = 1 WT mouse and 2 UCP1KO mice.**

Supplementary Figure 3: Mild impairment of respiratory chain in iBAT of UCP1KO mice housed at temperatures below their thermoneutral zone. Regulation of proteins related to lipid, Ca^2+^, and creatine metabolism in iBAT of WT and UCP1KO mice from a separate cohort housed at room temperature and in cold.

A) Protein expression of electron transport chain complexes in iBAT of C57BL/6J wild type (WT) and UCP1-knockout (UCP1KO) mice acclimated to different ambient temperatures: 30 °C “warm-acclimated” (WA), 20 °C “mild cold-acclimated” (MCA), or 6 °C “cold-acclimated” (CA), for at least three weeks. n = 4 – 5 animals (data from one experiment). “a” indicates a significant difference from the WA group of the respective genotype. “b” indicates a significant difference from the MCA group of the respective genotype. “c” indicates a significant difference between the two genotypes within one treatment level.

B) Schematic depiction of sample generation, processing, and subsequent proteome analysis. C57BL/6N WT and UCP1KO mice were acclimated to 23 °C or 5 °C. WT and UCP1KO iBAT samples were processed and analyzed together.

C) Regulation of proteins associated with lipid metabolism and D) futile Ca^2+^ and creatine cycling in iBAT of C57BL/6N WT and UCP1KO mice acclimated to 23 °C or 5 °C. The complete set of processed mass spectrometry data can be found in Supp. Table 4. n = 4 animals. Column labels represent individual mice: WT (WT23) and UCP1KO (UCP1KO23) mice acclimated to 23 °C, WT (WT5) and UCP1KO (UCP1KO5) mice acclimated to 5°C. Row labels represent gene symbols of proteins. Normalized protein intensities were scaled by calculating z-scores for each protein. Cell color indicates z-score. Abbreviations: Acetyl-CoA carboxylase alpha (Acaca), acetyl-CoA carboxylase beta (Acacb), ATP citrate lyase (Acly), acyl-CoA synthetase medium chain family member (Acsm), acyl-CoA synthetase short chain family member (Acss), CD36 molecule (Cd36), cell death inducing DFFA like effector a (Cidea), cell death inducing DFFA like effector c (Cidec), carnitine palmitoyltransferase 1A (Cpt1a), carnitine palmitoyltransferase 1B (Cpt1b), citrate synthase (Cs), ELOVL fatty acid elongase (Elovl), fatty acid binding protein (Fabp), fatty acid synthase (Fasn), lipin 1 (Lpin1), lipoprotein lipase (Lpl), perilipin (Plin), patatin like phospholipase domain containing (Pnpla), stearoyl-CoA desaturase 1 (Scd1), Fatp (Slc27a), ATPase sarcoplasmic/endoplasmic reticulum Ca^2+^ transporting (Atp2a), creatine kinase B (Ckb), creatine kinase, M-type (Ckm), creatine kinase, mitochondrial 2 (Ckmt2), inositol 1,4,5-trisphosphate receptor type (Itpr), ryanodine receptor 1 (Ryr1).

Supplementary Table 1:Expression of selected genes in eWAT and iBAT of C57BL/6J wild type (WT) and UCP1-knockout (UCP1KO) mice acclimated to different ambient temperatures: 30 °C “warm-acclimated” (WA), 20 °C “mild cold-acclimated” (MCA), or 6 °C “cold-acclimated” (CA), for at least three weeks. n = 4 – 5 animals (data from one experiment; confirmed in two independent experiments). “a” indicates a significant difference from the WA group of the respective genotype. “b” indicates a significant difference from the MCA group of the respective genotype. “c” indicates a significant difference between the two genotypes within one treatment level. Abbreviations : 18S ribosomal RNA (18Srna/Rn18s), ATP citrate lyase (Acly), aquaporin 7 (Aqp7), adipose triglyceride lipase also known as patatin-like phospholipase domain-containing protein 2 (Atgl/Pnpla2), cell death-inducing DNA fragmentation factor, alpha subunit-like effector A (Cidea), diacylglycerol O-acyltransferase 1 (Dgat1), diacylglycerol O-acyltransferase 2 (Dgat2), eukaryotic translation elongation factor 1 alpha 1 (Eef1a1), fatty acid synthase (Fas/Fasn), glycerol kinase (Gk), glycerol-3-phosphate dehydrogenase 1 (Gpd1), acyl-Coenzyme A dehydrogenase, long chain (Lcad/Acadl), lactate dehydrogenase A (Ldha), lactate dehydrogenase B (Ldhb), lipoprotein lipase (Lpl);, solute carrier family 16, member 1 (Mct1/Slc16a1), solute carrier family 16, member 3 (Mct2/ Slc16a3), pyruvate carboxylase (Pc), phosphoenolpyruvate carboxykinase 1, cytosolic (Pepck/Pck1), liver glycogen phosphorylase (Pygl), uncoupling protein 1 (Ucp1).

| Gene Name | WA | | | | | |  | MCA | | | | | |  | CA | | | | | |
| --- | --- | --- | --- | --- | --- | --- | --- | --- | --- | --- | --- | --- | --- | --- | --- | --- | --- | --- | --- | --- |
|  | *WT* | | | *UCP1KO* | | |  | *WT* | | | *UCP1KO* | | |  | *WT* | | | *UCP1KO* | | |
| **eWAT** |  |  |  |  |  |  |  |  |  |  |  |  |  |  |  |  |  |  |  |  |
| Lipolysis |  |  |  |  |  |  |  |  |  |  |  |  |  |  |  |  |  |  |  |  |
| *Atgl/Pnpla2* | 1.821 | ± | 0.149 | 1.786 | ± | 0.153 |  | 2.31 | ± | 0.123 | 3.087 | ± | 0.362^a^ |  | 3.465 | ± | 0.663^a^ | 5.922 | ± | 0.280^abc^ |
| *Cidea* | 0.002 | ± | 0 | 0.002 | ± | 0 |  | 0.014 | ± | 0.01 | 0.031 | ± | 0.017^a^ |  | 0.026 | ± | 0.009 | 0.082 | ± | 0.006^abc^ |
| DNL |  |  |  |  |  |  |  |  |  |  |  |  |  |  |  |  |  |  |  |  |
| *Fas/Fasn* | 3.255 | ± | 0.454 | 3.558 | ± | 0.639 |  | 6.001 | ± | 0.875^a^ | 9.888 | ± | 2.373^a^ |  | 15.479 | ± | 3.365^ab^ | 29.726 | ± | 1.137^abc^ |
| Glycerol-3-P synthesis | | |  |  |  |  |  |  |  |  |  |  |  |  |  |  |  |  |  |  |
| *Gk* | 0.031 | ± | 0.008 | 0.012 | ± | 0.002 |  | 0.033 | ± | 0.01 | 0.031 | ± | 0.009 |  | 0.039 | ± | 0.01 | 0.033 | ± | 0.004 |
| *Pc* | 4.828 | ± | 0.995 | 4.96 | ± | 0.722 |  | 7.644 | ± | 0.962 | 10.341 | ± | 2.186^a^ |  | 12.116 | ± | 2.045^a^ | 17.969 | ± | 2.909^abc^ |
| *Pepck/Pck1* | 1.593 | ± | 0.169 | 2.089 | ± | 0.48 |  | 1.471 | ± | 0.11 | 2.514 | ± | 0.349^c^ |  | 1.267 | ± | 0.197 | 2.51 | ± | 0.570^c^ |
| FA re-esterification | |  |  |  |  |  |  |  |  |  |  |  |  |  |  |  |  |  |  |  |
| *Dgat1* | 0.619 | ± | 0.058 | 0.764 | ± | 0.055 |  | 0.75 | ± | 0.028 | 0.834 | ± | 0.091 |  | 1.08 | ± | 0.128^ab^ | 1.522 | ± | 0.050^abc^ |
| *Dgat2* | 4.075 | ± | 0.995 | 5.054 | ± | 1.104 |  | 5.465 | ± | 1.025 | 4.148 | ± | 0.49 |  | 5.502 | ± | 0.679 | 7.054 | ± | 0.366 |
| Shutle |  |  |  |  |  |  |  |  |  |  |  |  |  |  |  |  |  |  |  |  |
| *Gpd1* | 0.756 | ± | 0.174 | 0.649 | ± | 0.051 |  | 0.917 | ± | 0.095 | 1.052 | ± | 0.16 |  | 1.333 | ± | 0.302 | 2.424 | ± | 0.274^abc^ |
|  |  |  |  |  |  |  |  |  |  |  |  |  |  |  |  |  |  |  |  |  |
| **iBAT** |  |  |  |  |  |  |  |  |  |  |  |  |  |  |  |  |  |  |  |  |
| Uncoupling of OXPHOS | | |  |  |  |  |  |  |  |  |  |  |  |  |  |  |  |  |  |  |
| *Ucp1* | 0.106 | ± | 0.04 | 0 | ± | 0 |  | 0.5 | ± | 0.094^a^ | 0.001 | ± | 0.000^c^ |  | 1.198 | ± | 0.173^ab^ | 0.001 | ± | 0.000^c^ |
| FA uptake and glycerol transport | | | |  |  |  |  |  |  |  |  |  |  |  |  |  |  |  |  |  |
| *Lpl* | 1.082 | ± | 0.046 | 1.27 | ± | 0.078 |  | 1.248 | ± | 0.083 | 2.086 | ± | 0.310^ac^ |  | 1.996 | ± | 0.096^ab^ | 2.833 | ± | 0.135^abc^ |
| *Cd36* | 0.431 | ± | 0.039 | 0.487 | ± | 0.023 |  | 0.603 | ± | 0.035^a^ | 0.627 | ± | 0.084 |  | 0.665 | ± | 0.042^a^ | 0.704 | ± | 0.047^a^ |
| *Aqp7* | 1.015 | ± | 0.107 | 1.007 | ± | 0.079 |  | 1.098 | ± | 0.069 | 1.049 | ± | 0.111 |  | 1.26 | ± | 0.169 | 1.069 | ± | 0.178 |
| Lipolysis |  |  |  |  |  |  |  |  |  |  |  |  |  |  |  |  |  |  |  |  |
| *Atgl/Pnpla2* | 2.399 | ± | 0.401 | 2.534 | ± | 0.31 |  | 3.825 | ± | 0.272^a^ | 3.507 | ± | 0.227 |  | 5.189 | ± | 0.655^ab^ | 2.973 | ± | 0.317^c^ |
| DNL |  |  |  |  |  |  |  |  |  |  |  |  |  |  |  |  |  |  |  |  |
| *Acly* | 1.204 | ± | 0.524 | 2.225 | ± | 0.805 |  | 7.651 | ± | 0.721^a^ | 8.067 | ± | 0.477^a^ |  | 9.151 | ± | 0.529^a^ | 3.936 | ± | 0.868^bc^ |
| *Fas/Fasn* | 3.299 | ± | 1.543 | 11.664 | ± | 5.033 |  | 26.003 | ± | 11.747 | 33.408 | ± | 3.47 |  | 49.653 | ± | 13.708^a^ | 29.427 | ± | 10.611 |
| FA oxidation |  |  |  |  |  |  |  |  |  |  |  |  |  |  |  |  |  |  |  |  |
| *Lcad/Acadl* | 5.763 | ± | 1.312 | 7.088 | ± | 1.027 |  | 14.85 | ± | 1.220^a^ | 11.901 | ± | 1.778 |  | 15.651 | ± | 1.544^a^ | 8.575 | ± | 1.351^c^ |
| Glycerol-3-P synthesis | | |  |  |  |  |  |  |  |  |  |  |  |  |  |  |  |  |  |  |
| *Gk* | 0.005 | ± | 0.001 | 0.006 | ± | 0.001 |  | 0.013 | ± | 0.002 | 0.02 | ± | 0.006^a^ |  | 0.026 | ± | 0.003^ab^ | 0.05 | ± | 0.004^abc^ |
| *Pc* | 1.383 | ± | 0.344 | 1.803 | ± | 0.358 |  | 2.914 | ± | 0.185^a^ | 2.213 | ± | 0.188 |  | 2.402 | ± | 0.322^a^ | 1.779 | ± | 0.228 |
| *Pepck/Pck1* | 2.934 | ± | 0.769 | 2.778 | ± | 0.304 |  | 3.326 | ± | 0.542 | 1.341 | ± | 0.334 |  | 2.659 | ± | 0.577 | 2.976 | ± | 0.499 |
| FA re-esterification | |  |  |  |  |  |  |  |  |  |  |  |  |  |  |  |  |  |  |  |
| *Dgat1* | 0.57 | ± | 0.103 | 0.703 | ± | 0.113 |  | 1.113 | ± | 0.022^a^ | 1.298 | ± | 0.177^a^ |  | 1.205 | ± | 0.082^a^ | 1.602 | ± | 0.033^ac^ |
| *Dgat2* | 2.276 | ± | 0.55 | 2.402 | ± | 0.379 |  | 4.043 | ± | 0.220^a^ | 2.336 | ± | 0.129^c^ |  | 4.097 | ± | 0.324^a^ | 2.414 | ± | 0.183^c^ |
| Lactate metabolism | |  |  |  |  |  |  |  |  |  |  |  |  |  |  |  |  |  |  |  |
| *Ldha* | 2.82 | ± | 0.902 | 2.806 | ± | 0.562 |  | 5.435 | ± | 0.306^a^ | 5.582 | ± | 0.617^a^ |  | 8.671 | ± | 0.632^ab^ | 2.996 | ± | 0.746^bc^ |
| *Ldhb* | 0.974 | ± | 0.342 | 0.764 | ± | 0.237 |  | 2.206 | ± | 0.125^a^ | 1.505 | ± | 0.378 |  | 2.875 | ± | 0.284^a^ | 1.121 | ± | 0.233^c^ |
| *Mct1/Slc16a1* | 0.417 | ± | 0.115 | 0.356 | ± | 0.06 |  | 0.739 | ± | 0.067^a^ | 0.907 | ± | 0.157^a^ |  | 1.516 | ± | 0.094^ab^ | 0.724 | ± | 0.133^c^ |
| *Mct4/Slc16a3* | 0.004 | ± | 0.002 | 0.004 | ± | 0.001 |  | 0.005 | ± | 0 | 0.004 | ± | 0.001 |  | 0.006 | ± | 0.001 | 0.003 | ± | 0.001 |
| Shuttle |  |  |  |  |  |  |  |  |  |  |  |  |  |  |  |  |  |  |  |  |
| *Gpd1* | 1.748 | ± | 0.606 | 1.828 | ± | 0.384 |  | 6.585 | ± | 0.285^a^ | 9.352 | ± | 1.315^ac^ |  | 10.146 | ± | 0.9753^ab^ | 3.14 | ± | 0.525^bc^ |
| Degradation of glycogen | | |  |  |  |  |  |  |  |  |  |  |  |  |  |  |  |  |  |  |
| *Pygl* | 1.255 | ± | 0.565 | 1.111 | ± | 0.332 |  | 4.077 | ± | 0.481^a^ | 3.117 | ± | 0.740^a^ |  | 4.136 | ± | 0.256^a^ | 1.067 | ± | 0.134^bc^ |
